# Stress-relaxing granular bioprinting materials enable complex and uniform organoid self-organization

**DOI:** 10.1038/s41563-026-02519-4

**Published:** 2026-03-10

**Authors:** Austin J. Graham, Michelle W. L. Khoo, Vasudha Srivastava, Sara Viragova, Honesty Kim, Kavita Parekh, Kelsey M. Hennick, Malia Bird, Nadine Goldhammer, Jie Zeng Yu, Grace Hu, Natasha T. Brinkley, Lucas Pardo, Jasmine S. Amaya, Cameron D. Morley, Nishant Chadha, Paul Lebel, Sanjay Kumar, Jennifer M. Rosenbluth, Tomasz J. Nowakowski, Ovijit Chaudhuri, Ophir Klein, Rafael Gómez-Sjöberg, Zev J. Gartner

**Affiliations:** 1 Department of Pharmaceutical Chemistry, University of California San Francisco, San Francisco, CA, USA; 2 Chan Zuckerberg Biohub SF, San Francisco, CA, USA; 3 Department of Orofacial Sciences, University of California San Francisco, San Francisco, CA, USA; 4 Department of Bioengineering, University of California Berkeley, Berkeley, CA, USA; 5 Weill Institute for Neurosciences, University of California San Francisco, San Francisco, CA, USA; 6 Department of Medicine, University of California San Francisco, San Francisco, CA, USA; 7 Graduate Program in Bioengineering, University of California San Francisco and University of California Berkeley, San Francisco, CA, USA; 8 Department of Chemical and Biomolecular Engineering, University of California Berkeley, Berkeley, CA, USA; 9 Department of Bioengineering and Therapeutic Sciences, University of California San Francisco, San Francisco, CA, USA; 10 Department of Mechanical Engineering, Stanford University, Stanford, CA, USA; 11 Department of Bioengineering, Stanford University, Stanford, CA, USA; 12 Department of Pediatrics, Cedars-Sinai Guerin Children’s, Los Angeles, CA, USA; 13 Center for Cellular Construction, University of California San Francisco, San Francisco, CA, USA

## Abstract

Complex and robust tissue self-organization requires defined initial conditions and dynamic boundaries—neighbouring tissues and extracellular matrix that actively evolve to guide morphogenesis. A major challenge in tissue engineering is identifying material properties that are compatible with controlling initial culture conditions while mimicking dynamic tissue boundaries. Here we describe a highly tunable granular biomaterial, MAGIC matrix, that supports both long-term bioprinting and gold-standard tissue self-organization. We identify that significant stress relaxation at the long timescales and large deformation magnitudes relevant to self-organization is required for optimal morphogenesis. We apply optimized MAGIC matrices toward precise extrusion bioprinting of saturated cell suspensions directly into three-dimensional culture. Carefully controlling initial conditions for tissue growth yields dramatic increases in organoid reproducibility and complexity across multiple tissue types, enabling high-throughput generation of organoid arrays and perfusable three-dimensional microhysiological systems. Our results identify key biomaterial parameters for optimal organoid morphogenesis and lay the foundation for fabricating more complex and reproducible self-organized tissues.

Controlling the uniformity and complexity of tissues grown in vitro remains a major challenge^1,2^. Tissues in vivo typically self-organize juxtaposed to other tissues that act as living boundaries, changing shape and composition in parallel to direct morphogenesis^3^. Guiding tissue self-organization in vitro requires replicating the important functional characteristics of these living interfaces, and satisfying these requirements remains a major challenge in materials science and tissue engineering^4^. Non-living biomaterials attempt to replicate key chemical and rheological cues derived from living boundaries, with gold standards including reconstituted basement membrane matrices (rBMs) such as Matrigel^5^. For rBMs, recent studies have highlighted the presence of laminin epitopes and soft viscoelastic rheological properties as essential for epithelial polarity and morphogenesis^6,7^. This has enabled the design of synthetic biomaterials that support symmetry breaking during self-organization^1,8–13^. However, the quantitative relationships between biomechanical properties and outcomes ofmorphogenesis remain unclear.

An additional challenge to directing the uniform and complex self-organization of tissues in vitro is setting the initial conditions of a culture—defined here as the total number of cells, the proportions ofeach cell type and their positions in three-dimensional (3D) space. In vivo, each stage in morphogenesis emerges from a limited set of initial conditions, and large changes in initial conditions can have a profound impact on growth outcomes^2^. To control initial conditions engineers have developed platforms such as microwell arrays, microphysiological systems and 3D bioprinting that can be deployed together with biomaterials to further constrain self-organization^14–16^. Typically, however, these engineered systems are incompatible with the optimal rheological properties for tissue morphogenesis, requiring that tissues are transferred between different materials after fabrication (for example, microwells) or that their structure remains static within the device (for example, microphysiological systems).

Among engineering platforms, embedded 3D bioprinting of saturated cell suspensions provides remarkable flexibility for ‘writing’ the initial conditions of tissue morphogenesis^17–20^. However, much of biomaterial design for 3D printing has focused on optimizing complex geometries while sustaining cell viability and simple behaviours such as growth and motility. When the goal is to build tissues, however, active mechanical processes such as lumenization, inflation, compaction, cell sorting and folding that contribute to the final tissue form are equally important^1 ,21,22^ (Fig. 1a). Although rBMs support these behaviours, and Matrigel has been used as an embedded bioprinting material^17^, they have several critical limitations preventing generation of tissues of high complexity or in high throughput.

As an alternative, granular microgels are frequently applied as embedded 3D bioprinting materials because they can support the structure of extruded materials after printing^23,24^. A critical feature ofthese materials is reversible yield-stress behaviour, in which the granular microgels yield in response to the printhead and extruded material, then recover to provide elastic support to the bioink. However, most granular materials are not optimal for long-term cell health, and little is known about whether the properties necessary for bioprinting are compatible with complex tissue morphogenesis^25^. Several groups have introduced interstitial matrices derived from natural extracellular matrix (ECM) such as collagen I to improve cell survival and dynamics^26–28^. However, the rheological properties and chemical composition ofthe resulting materials can limit self-organization. For example, collagen I-containing ECM is not optimized to support epithelial growth and morphogenesis compared to rBMs^17^.

In this study, we quantify the relationship between granular biomaterial rheology and tissue self-organization to reveal that when controlling for modulus at short timescales, the rate and extent of stress relaxation at long timescales determines the quantitative outcome oftissue morphogenesis. We apply this insight to design an optimized embedded bioprinting material termed Matrigel-Alginate Granular-Interstitial Composite (MAGIC) matrix. MAGIC matrices employ alginate microgels that are optically transparent and approximately cell-sized, facilitating yielding behaviour, high-fidelity embedded bioprinting, imaging and organoid growth. Utilizing Matrigel as the interstitium creates a composite matrix that fluidizes upon shear at 4 °C to allow for long print times (≥2 h), but cross-links at 37 °C to create an environment with similar rheology to pure Matrigel across several metrics. We find that MAGIC matrices are remarkably tunable across properties including yield-stress, shear modulus and stress relaxation at short and long timescales. To capitalize on the reproducibility, scalability and automation afforded by 3D printing into MAGIC matrices, we designed a piezoelectric bioprinting platform that allows for full *xyz* control, real-time imaging and flexible print geometries to control the initial conditions of organoid self-organization. Together, MAGIC matrix bioprinting led to nearly 100% organoid formation efficiency, dramatic improvements in interorganoid homogeneity, orders-of-magnitude improvement in the statistical power of phenotypic assays, and enabled patterning of perfusable 3D microphysiological systems.

## Results

### MAGIC matrices can be tuned for optimal 3D patterning at 4 °C

**Extended Data Fig. 1 |:**
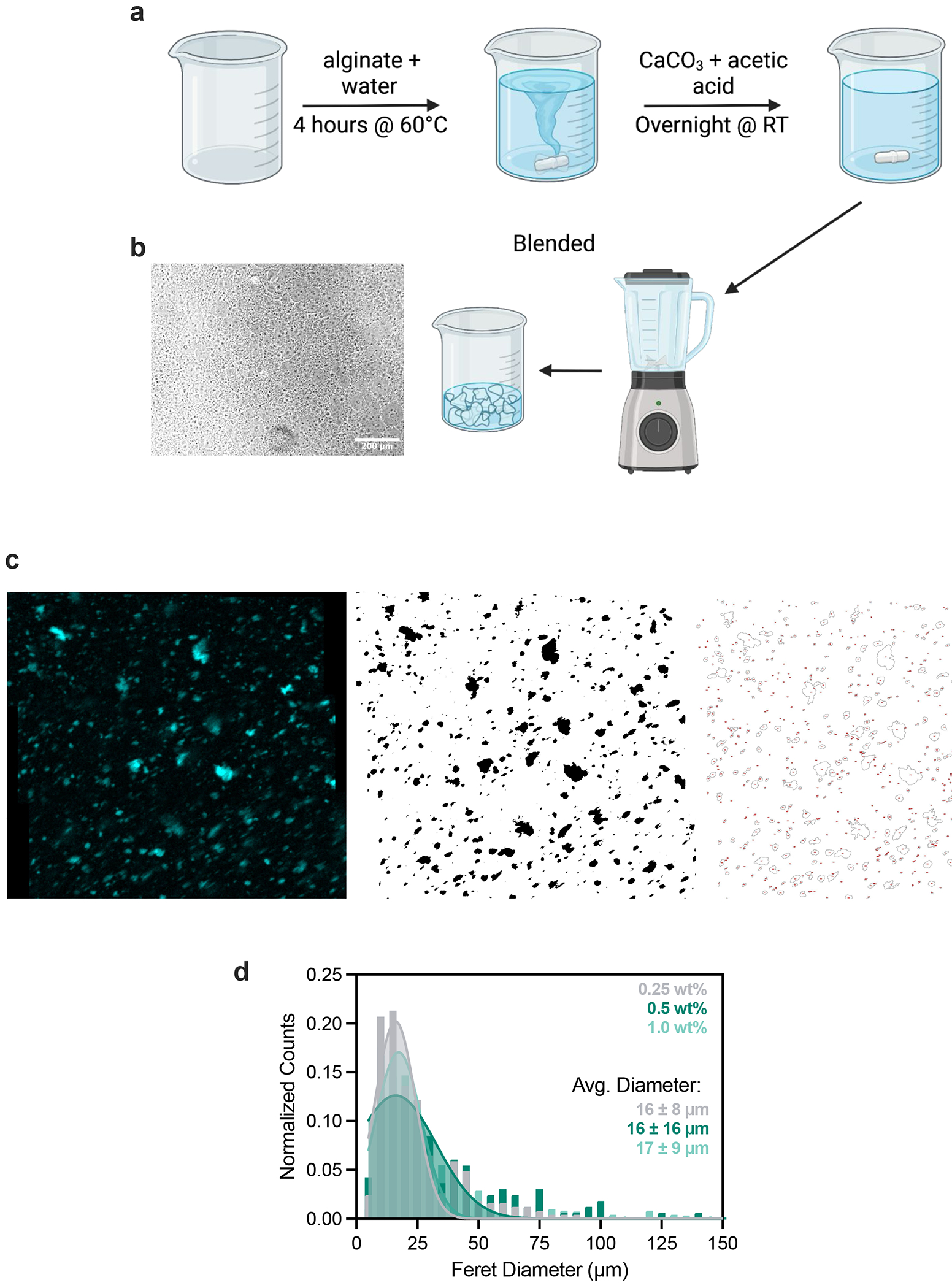
Alginate microgels are roughly cell-sized. **a**. Cartoon workflow for preparation of alginate microgel slurry. **b**. Bright-field image of microgel slurry after synthesis, with nearly transparent microgels. Scale bar = 200 μm. **c**. Representative images outlining workflow for quantifying microgel size. The polyanionic alginate backbone was positively stained with DAPI and segmented in Fiji to calculate particle diameter. **d**. Distribution of alginate microgel sizes fit to a Gaussian distribution. Data shown are mean ± SD from *n* = 3 separate images from 3 separate fields of view for each alginate wt% and 3 independent AMG preparations.

**Fig. 1 |:**
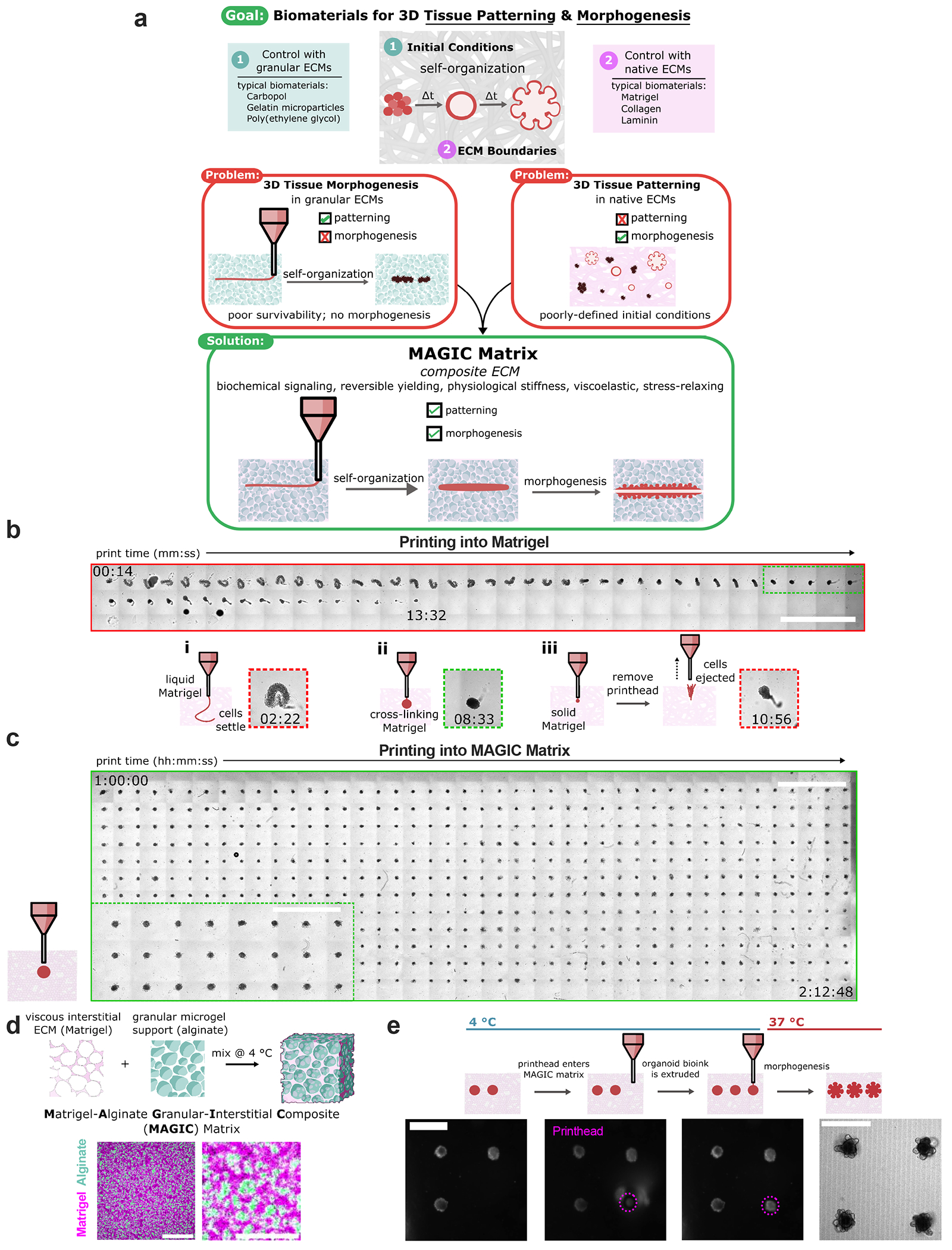
MAGIC extracellular matrices are embedded bioprinting materials that enable both patterning and morphogenesis of organoids. **a**, Biomaterials designed for tissue patterning, such as some hydrogel microparticles, fail to support long-term tissue health or complex behaviours such as morphogenesis. Biomaterials typically used for such behaviours include Matrigel and collagen, but these materials do not mechanically support embedded bioprinting. MAGIC matrices exhibit a suite of biochemical and mechanical behaviours that, in sum, enable both complex patterning and morphogenesis of organoid bioinks. **b**, Organoid slurries printed into Matrigel while cross-linking at room temperature first distort and settle to the bottom of the dish (1) due to insufficient mechanical support, followed by a narrow regime (~2 min) during which printing is viable (2), and eventually are ejected or excluded as the hydrogel sets (3). Insets: examples of each regime. Scale bar, 4 mm. **c**, Organoid bioink printed into MAGIC matrix at 4 °C conforms to the desired morphology due its reversible yield-stress properties, enabling printing of many organoids (*n* = 528) over long times (*t* > 2 h). MAGIC matrix was left in chilled conditions on the printer for 1 h before printing to demonstrate printing longevity. Scale bar, 4 mm. Inset: zoomed view of organoids printed in MAGIC matrix. Scale bar, 2 mm. **d**, MAGIC matrix comprises of an inert alginate microgel granular support and a viscous basement membrane interstitium. Confocal microscopy images showing a standard MAGIC matrix composition using FITC-alginate microgels and NHS-labelled Matrigel. Scale bar, 100 μm. Zoomed view shows white pixels where fluorescent signals overlap, suggesting ECM in alginate microgels. Scale bar, 40 μm. **e**, Sequential bright-field images demonstrating MAGIC matrix bioprinting, in which the printhead enters cold MAGIC matrix, extrudes organoid slurry and is then removed from the matrix, leaving a geometrically and spatially controlled feature behind. These organoid bioinks subsequently undergo self-organization at 37 °C to form mature organoids. Scale bars, 500 μm. Diagram in **d** created in BioRender; Graham, A. https://BioRender.com/chloe1z (2026).

Matrigel is the gold-standard biomaterial for supporting the complex morphogenesis of epithelial organoids. However, it does not support long bioprinting windows (Supplementary Movie 1). When extruding a cell-dense bioink as it warms from 4 °C to room temperature (as previously described^17^), Matrigel is initially too liquid to support printed cells and filaments settle to the bottom of the dish after extrusion (Fig. 1b.1). During a narrow span of ~2 min during gelation, the bath supports printed shapes (Fig. 1b.2), after which the gel became too elastic and cells are not extruded or ejected from the printing plane (Fig. 1b.3). We therefore sought to design a reversible yield-stress granular material that supported embedded bioprinting at 4 °C but could be tuned to match the rheological properties of biomaterials such as Matrigel at 37 °C. For the granular phase we turned to alginate as an optically transparent biomaterial that is largely inert in mammalian culture and has been used in both bioprinting and organoid culture applications^26,29^. We used microgels that were approximately cell sized to facilitate self-organization, migration and diffusion^27^ (Extended Data Fig. 1). We supplemented the microgels with an interstitial matrix of Matrigel because its rich chemical composition is necessary to support morphogenesis of most epithelial organoids. At 4 °C, this material supported bioprinting of cell-dense bioinks for >2 h without compromising print fidelity (Fig. 1c). We termed this class of composite materials MAGIC matrix (Fig. 1d,e).

**Extended Data Fig. 2 |:**
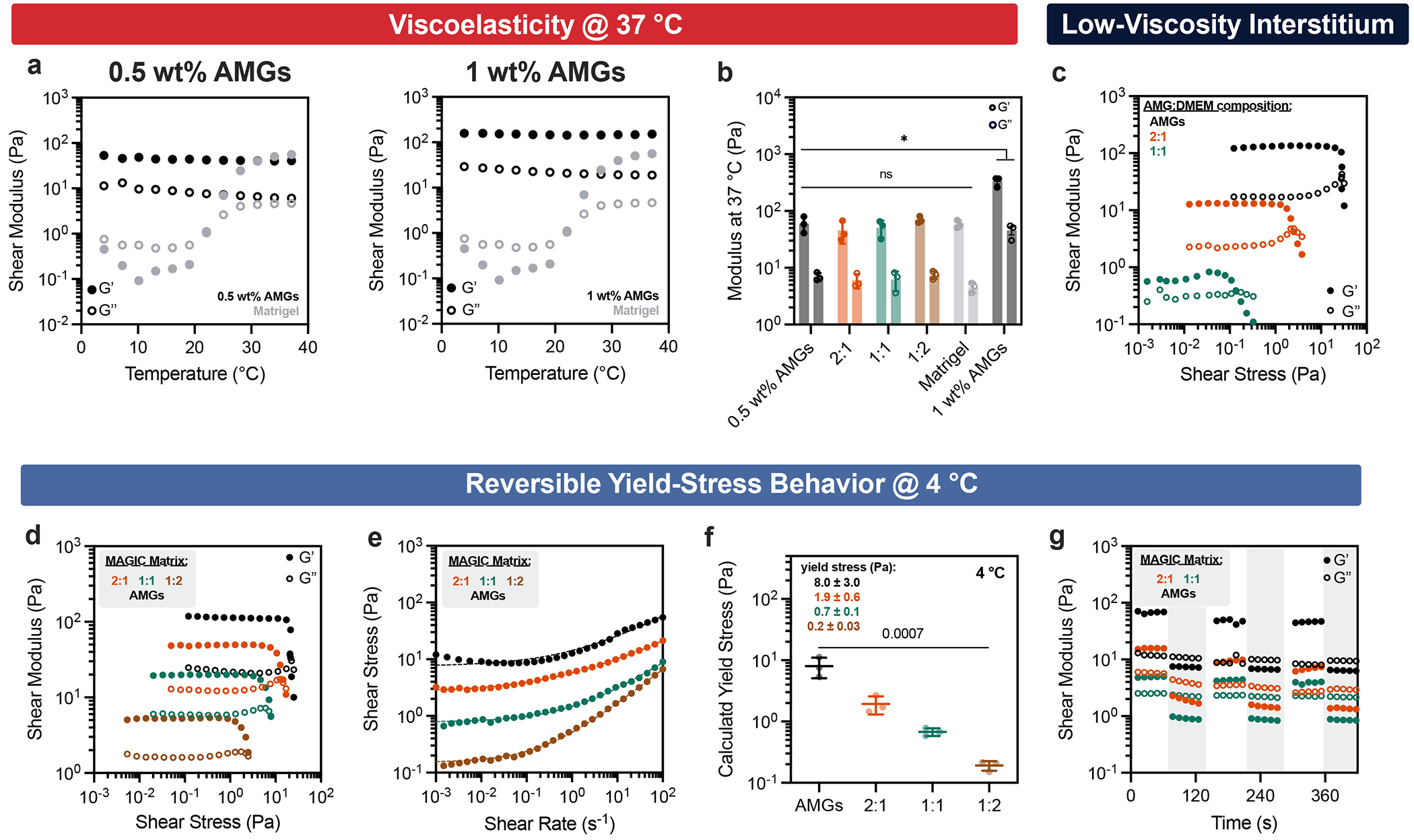
MAGIC matrices are tunable embedded printing and cell culture biomaterials. **a**. Storage and loss modulus of (left) 0.5 wt% and (right) 1 wt% alginate microgel (AMG) pelleted slurry and Matrigel as a function of temperature. **b**. Storage and loss moduli of MAGIC matrix formulations at 37 °C using pelleted 0.5 wt% AMGs or (right) 1 wt% AMG slurry at 1 Hz and 1% strain. Data shown are mean ± SD from n = 3 independent microgel preparations. * = p < 0.05 for both storage and loss modulus of all MAGIC matrix formulations and 1 wt% AMG slurry compared to pure Matrigel as determined by one-way ANOVA with Dunnett’s multiple comparisons. **c**. Oscillatory amplitude sweeps at 4 °C for various compositions of pelleted AMGs or AMGs diluted in cell culture media as a low-viscosity interstitium compared to Matrigel, which decreases jamming and yield-stress. 1:2 AMG:DMEM compositions did not exhibit yielding behaviour. **d**. Oscillatory amplitude sweeps at 4 °C for various MAGIC matrix compositions show yielding behaviour indicated by G’ and G” cross-over. Data shown are representative of n = 3 independent microgel preparations. **e**. Unidirectional shear rate measurements at 4 °C fit to a Herschel–Bulkley power-law model. **f**. MAGIC matrix yield stress values calculated using Herschel–Bulkley fits in (D). Data shown are mean ± SD of n = 3 independent microgel preparations; statistical significance was determined by one-way ANOVA with P shown. **g**. Reversible yield-stress test wherein applied strain is alternated between 1% and 100% for a variety of MAGIC matrix formulations at 4 °C. Grey bars indicate areas of 100% strain. Cross-over and recovery of G’ and G” indicates reversible viscoelastic behaviour. For **d, e**, and **g**, data are representative of *n* = 3 independent microgel preparations.

**Fig. 2 |:**
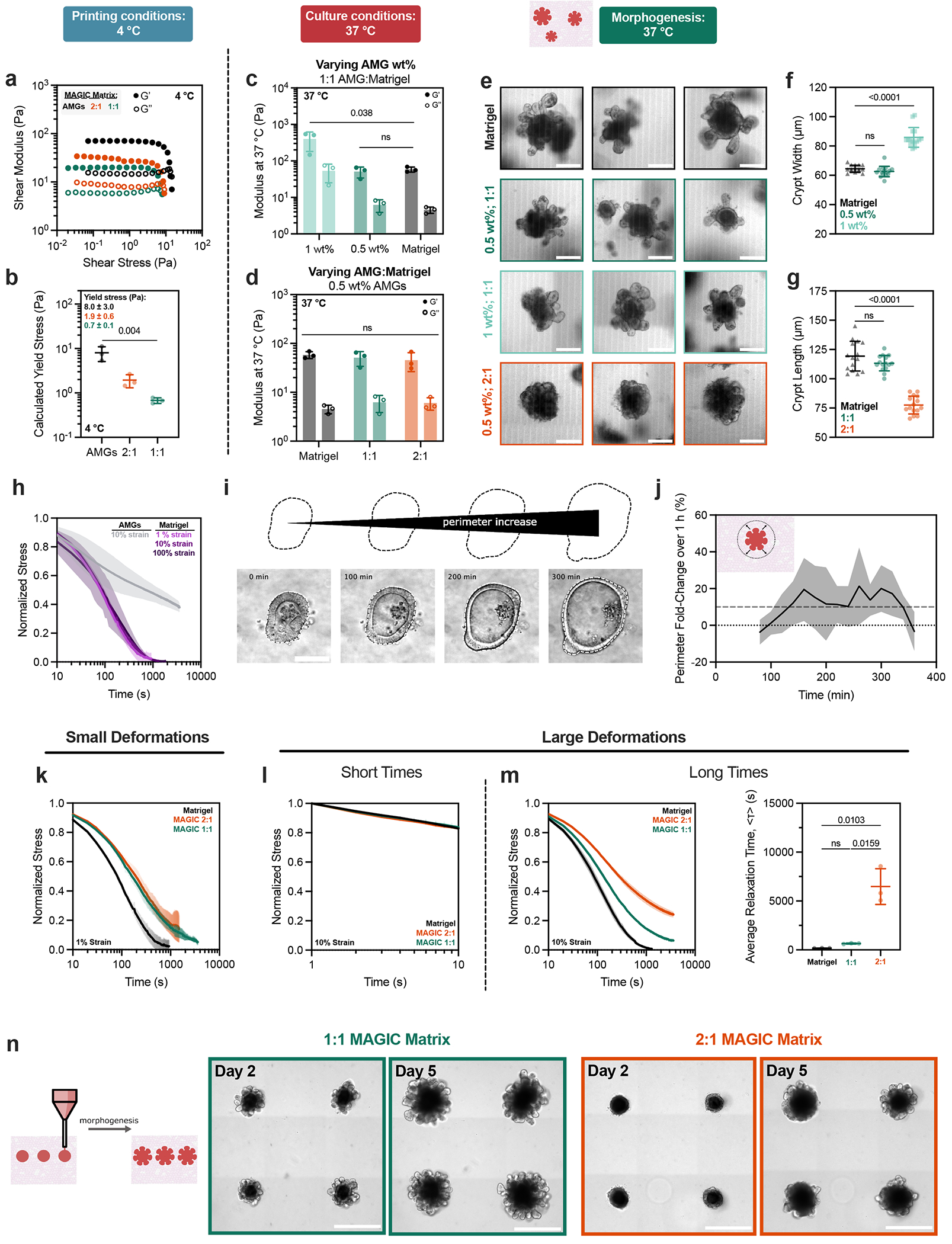
Rheological properties of MAGIC matrices including low stiffness and stress relaxation drive gold-standard morphogenesis. **a**, At 4 °C, oscillatory amplitude sweeps at 1 Hz reveal that various MAGIC matrix compositions behave as yield-stress materials, indicated by *G*′ and *G*″ cross-over. **b**, MAGIC matrices behave as Herschel–Bulkley fluids at 4 °C with yield-stresses calculated using a power-law model. **c**, Storage and loss moduli at 1 Hz and 1% strain of MAGIC matrices at 37 °C prepared from 1 wt% or 0.5 wt% AMGs. **d**, Storage and loss moduli at 1 Hz and 1% strain of MAGIC matrices at 37 °C prepared using different volume ratios of 0.5 wt% AMGs:Matrigel. **e**, Mouse intestinal organoids grown in pure Matrigel (top row) and MAGIC matrices of several compositions (lower rows) 5 days after seeding. Scale bars, 200 μm. **f**, Quantification of organoid crypt width as a function of matrix composition. **g**, Quantification of crypt length as a function of matrix composition. **h**, Normalized stress-relaxation curves for undiluted 0.5 wt% AMG slurry at 10% strain or Matrigel over 1 h at 1%, 10% or 100% strain. **i**, Bright-field images and segmented cartoons simulating organoid growth over time in Matrigel. Scale bar, 100 μm. **j**, Measurement of material strain at the organoid–ECM interface over time quantified using segmented organoid perimeters. The dashed line at 10% strain represents the strain value used for most stress-relaxation measurements. **k**, Normalized stress-relaxation curves for Matrigel or MAGIC matrices over 1 h at 1% strain. **l**, Normalized stress-relaxation curves for Matrigel or MAGIC matrices over 10 s at 10% strain. **m**, Normalized stress-relaxation curves for Matrigel or MAGIC matrices over 1 h at 10% strain (left) and quantification of average relaxation time for each matrix using a stretched exponential model (right). **n**, Organoid array bioprinting experiments demonstrating similar impacts on crypt morphogenesis when MAGIC matrix compositions are used as bioprinting support baths. Scale bars, 500 μm. For all rheological experiments, data shown are mean ± s.d. from *n* = 3 independently prepared replicates. For crypt length and width measurements, data shown are mean ± s.d. on the median *n* = 15 crypts from ≥10 organoids per matrix condition. For ECM strain during organoid growth, data shown are mean ± s.d. of *n* = 9 organoids. Statistical significance was determined by one-way ANOVA with Tukey’s multiple comparisons (**b,c,m**) or Dunnett’s multiple comparison (**f,g**); values shown for *P* determined by one-way ANOVA (**b**) or by multiple comparisons (**c,m,f,g**) < 0.05; NS, not significant.

The composite biomaterial was tunable along several rheological parameters and in different temperature regimes to support bioprinting and tissue morphogenesis. To demonstrate this tunability we prepared several compositions and characterized their properties by shear rheology at 4 °C (relevant to bioprinting) and 37 °C (relevant to organoid culture). Preparations included undiluted jammed alginate microgels (AMGs); three different volume fractions ofAMG slurry diluted in Matrigel at 2:1, 1:1 and 1:2 AMGs:Matrigel by added volume; and two different polymer weight fractions in the AMG preparation at 0.5 and 1 wt%. All compositions of MAGIC matrix demonstrated reversible yield-stress behaviour at 4 °C and fitted well to a Herschel–Bulkley exponential model, suggesting their utility as embedded bioprinting materials (Fig. 2a). Varying MAGIC matrix composition tuned yield-stress values over an order of magnitude (Fig. 2b). Viscoelasticity and yield-stress were increased when using a viscous interstitial material such as Matrigel as opposed to cell culture media (Extended Data Fig. 2), highlighting the importance of the interstitial phase in granular materials at 4 °C (ref. 30).

### Stress relaxation over length- and time-scales relevant to morphogesis drive crypt self-organization

To assess MAGIC matrix behaviour under physiological conditions, we next measured viscoelastic properties following cross-linking at 37 °C. At 1 wt% alginate, the storage and loss moduli of the composite matrix were significantly greater compared with pure Matrigel (Fig. 2c). In contrast, at 0.5 wt% alginate, moduli of the composite matrix were similar to that of pure Matrigel across a range of compositions (Fig. 2d). These analyses suggested that 0.5 wt% MAGIC matrices had similar rheological properties to Matrigel, and therefore, may equivalently support tissue growth and morphogenesis.

**Extended Data Fig. 3 |:**
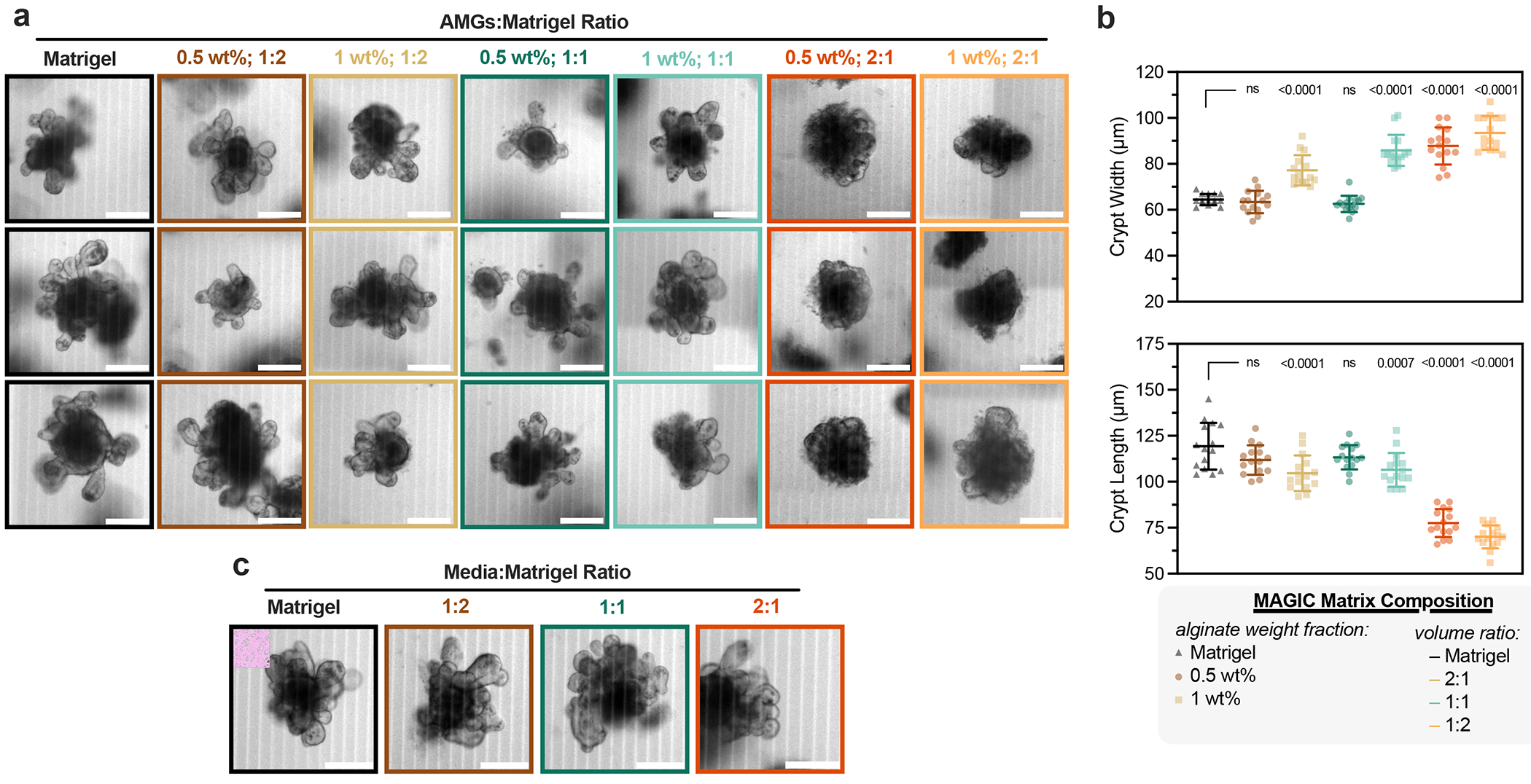
MAGIC matrix composition impacts organoid morphogenesis. **a**. Representative images of mouse intestinal organoids at 5 days after manually seeding into Matrigel and MAGIC matrices of varying AMG wt% and composition with Matrigel. MAGIC matrix compositions are represented as added volume ratio of AMGs:Matrigel. Scale bars = 200 μm. **b**. Quantification of organoid crypt width (top) and crypt length (bottom) as a function of matrix composition. Data shown are mean ± SD on the median n = 15 crypts from ≥10 organoids per matrix condition. Statistical significance was determined by one-way ANOVA with Dunnett’s multiple comparison; values shown for p < 0.05; ns = not significant. **c**. Representative images of organoids grown in Matrigel diluted at various volume ratios with mouse intestinal organoid growth medium. Scale bars = 200 μm.

**Extended Data Fig. 4 |:**
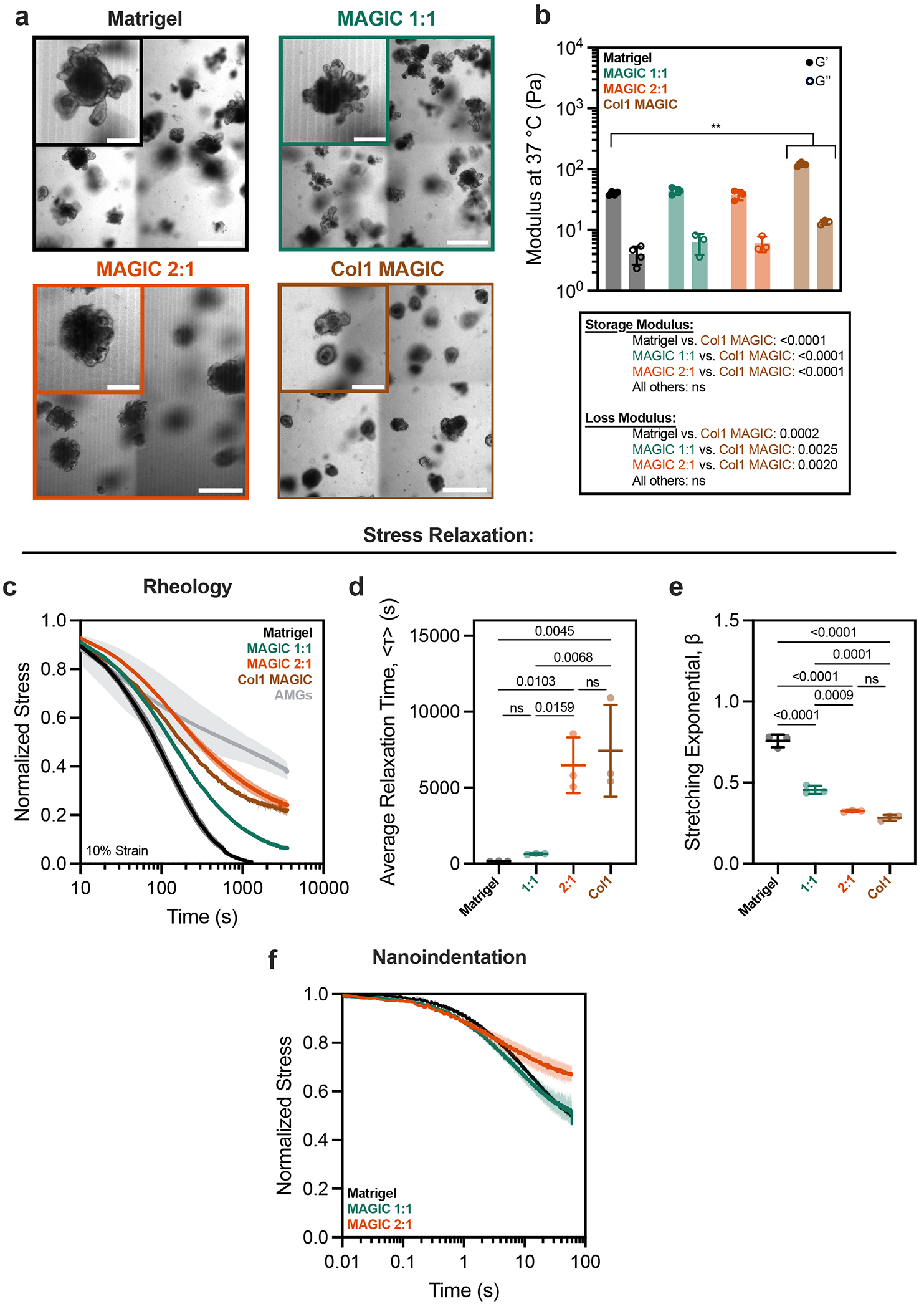
ECM stiffness and stress relaxation determine phenotypes of crypt morphogenesis in intestinal organoids. **a**. Representative images of mouse intestinal organoids at 5 days after manually seeding into Matrigel, MAGIC matrices, and collagen 1-containing matrices. Collagen 1-containing matrices were composed of 25% 4 mg/mL Bovine Col-1, 25% Matrigel, and 50% pure AMGs by added volume (final concentration of 1 mg/mL Col-1). Scale bars = 500 μm. Insets show zoomed in view of representative organoids. Scale bars = 200 μm. **b**. Storage and loss moduli of Matrigel, MAGIC matrix, and Collagen I-containing matrices at 37 °C, 1 Hz, and 1% strain. Table summarizes statistical analysis. c. Normalized stress-relaxation curves for Matrigel, AMGs, MAGIC matrices, and Collagen 1-containing matrices over 1 h at 10% strain. **d, e**. Quantification of average relaxation time (**d**) and stretching exponential (**e**) for each matrix determined using a stretched exponential model. **f**. Normalized stress-relaxation measurements of Matrigel and MAGIC matrices of various compositions using nanoindentation at 50 μm indentation for 60 s. Data shown are mean ± SD from n = 3 independent ECM preparations. Statistical significance was determined by one-way ANOVA with Tukey’s multiple comparisons with p values shown; ** = p ≤ 0.01; ns = not significant.

We evaluated the performance of these ECMs in morphogenesis assays using mouse duodenal organoids. Intestinal organoid crypt budding and lumen expansion is highly sensitive to matrix mechanics^5,31–33^. We therefore measured crypt width and length across all matrix formulations in manual dome culture (Fig. 2e). All organoids in MAGIC matrices containing 1 wt% AMGs formed shorter and wider crypts than Matrigel controls, consistent with their increased stiffness (Extended Data Fig. 3). Similarly, composite matrices formulated with both with 25% Matrigel and 1 mg ml^−1^ collagen I were significantly stiffer than 0.5 wt% MAGIC matrices and gave rise to organoids with minimal or no crypts^26^ (Extended Data Fig. 4). In contrast, MAGIC matrix compositions with 0.5 wt% at 50% AMG volume fraction or lower were indistinguishable from those grown in pure Matrigel (Fig. 2f).

We were surprised to find that formulations of MAGIC sharing 0.5 wt% microgels but having different volume fractions of Matrigel had different crypt morphologies despite their nearly identical storage and loss moduli. Specifically, 2:1 AMGs:Matrigel had significantly shorter and wider crypts compared with 1:1 MAGIC and Matrigel (Fig. 2g and Extended Data Fig. 3). These findings were not a consequence of Matrigel dilution, indicating that potential differences in the concentration of the interstitial ECM did not contribute to these observations (Extended Data Fig. 3). We noted that intestinal organoids undergo large morphological changes over hour timescales during morphogenesis, including crypt budding and lumen inflation^33^, which induce large mechanical strains in the surrounding hydrogel. Additionally, stress relaxation over these timescales affects single-cell behaviours^34–36^ and is required for symmetry-breaking events at the tissue scale ^7,37–40^. Thus, we hypothesized that crypt morphology may be quantitatively linked to stress-relaxation rate and magnitude at long timescales and large strains, even when the response of the materials at short length scales and timescales was identical. This hypothesis was not previously testable because previous investigations of stress relaxation used materials whose stress-relaxation rate was correlated with loss tangent^34–38^.

To test this hypothesis, we first measured stress relaxation of Matrigel over 1 h at variable strain. Notably, Matrigel completely relaxed accumulated stresses in ~15 min for a variety of strain magnitudes, whereas undiluted AMGs did not (Fig. 2h). We next investigated the relevant length scale of stress relaxation for intestinal organoid morphogenesis by quantifying organoid perimeter growth over time^41^. Over 1-h segments we observed an average perimeter expansion of ~10% (Fig. 2i,j). Importantly, these changes in organoid size occur over timescales much longer than standard biomaterial measurement regimes (~1 Hz).

We then compared stress-relaxation properties of 1:1 and 2:1 MAGIC matrix formulations given that these compositions had matched moduli but exhibited striking differences in crypt morphology. For small deformations (approximated as 1% strain), MAGIC matrices dissipated stress nearly identically over both short and long timescales (Fig. 2k). Although both formulations dissipated stress more slowly than pure Matrigel, they ultimately dissipated 80–90% of accumulated stress after 1 h. For larger deformations relevant to organoid morphogenesis (10% strain), stress relaxation of both MAGIC formulations and Matrigel occurred nearly identically at short time-scales (≤10 s) (Fig. 2l). However, over longer times (10% strain over 1 h), 2:1 MAGIC matrices relaxed stress significantly more slowly than both Matrigel and 1:1 MAGIC, providing a potential explanation for their differential effects on organoid morphogenesis (Fig. 2m). We quantified this effect using a stretched exponential function to calculate average relaxation time for each composition^36^ (Fig. 2m and Supplementary Information, section 2). Critically, 1:1 MAGIC matrices dissipated ~95% of internal stresses after 1 h, with as little as ~0.2 Pa of residual stress remaining in the material. This bulk property of the materials was further explored at the micrometre scale by performing nanoindentation to measure stress relaxation, where we observed impeded relaxation in 2:1 MAGIC matrix compared with 1:1 MAGIC and Matrigel (Extended Data Fig. 4). Notably at this scale, 1:1 MAGIC matrix and Matrigel relaxed stress at a similar rate and magnitude.

To establish that these properties of MAGIC matrix translated to a 3D bioprinting context, we used a bioink derived from a saturated suspension of dissociated intestinal organoids (see next section). Crypts appeared more slowly in 2:1 MAGIC matrix than in 1:1 matrix, suggesting that the rate of crypt morphogenesis may also be impacted by stress relaxation (Fig. 2n). Taken together, these results demonstrate that for materials matched in viscoelasticity at short length scales and timescales, differences in mechanical properties at long timescales and large strains can profoundly impact complex biological processes such as morphogenesis.

### A piezoelectric printhead enables precise, automated direct ‘writing’ of dense cell slurry bioinks

**Fig. 3 |:**
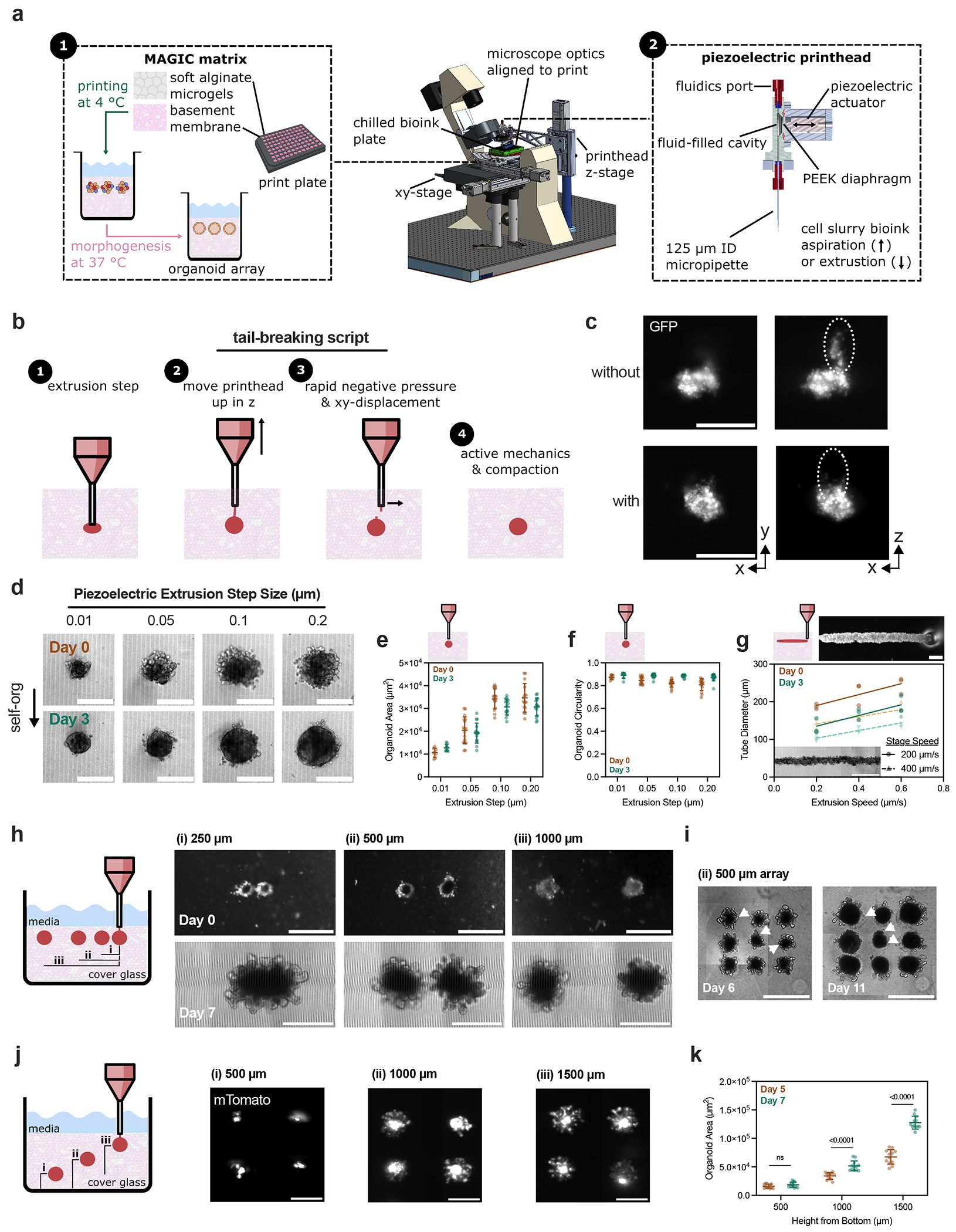
MAGIC matrix bioprinting utilizes a piezoelectric printhead to precisely aspirate and extrude cell slurry bioinks and can be programmed to generate organoid arrays. **a**, Schematic illustrating the MAGIC matrix bioprinting platform, including the benefits of MAGIC matrix (left) and the piezoelectric printhead (right). **b**, The piezoelectric printhead and precise *xyz* control tolerate rapid pressure ramps and print plate movement, enabling scripts such as tail-breaking to improve print fidelity of viscous cell slurry bioinks. **c**, Representative fluorescent images of bioprinted spheroids with and without a tail-breaking script enabled by piezoelectric bioprinting through rapid changes in applied voltage and printhead position. Scale bars, 200 μm. **d**, Representative bright-field images of bioprinted Caco-2 tissues at day 0 and day 3 post-print as a function of extrusion step size controlled via applied voltage. Data are representative of at least *n* = 9 individual tissues per extrusion step condition. Scale bars, 200 μm. **e,f**, Organoid area (**e**) and circularity (**f**) measured using maximum intensity projections of confocal *z*-stack images of GFP-expressing Caco-2 cell slurries. At both day 0 and day 3, organoid area significantly depends on extrusion step size until beyond 1.0 μm as determined by one-way ANOVA with Tukey’s multiple comparisons. Data shown are mean ± s.e.m. of *n* ≥ 9 individual tissues per extrusion condition. At day 3, organoid circularity does not depend significantly on extrusion step size as determined by one-way ANOVA. **g**, Quantification of bioprinted tube diameter at day 0 and day 3 postprinting as a function of both stage translation speed and extrusion step speed. Fit demonstrates that initial and final tube diameter are approximately linear functions of extrusion step speed for a given stage translation speed. Above, representative bright-field image of organoid tube during print. Scale bar, 200 μm. Inset: representative tube exhibiting signs of lumenization 3 days after printing. Scale bar, 500 μm. Data shown are *n* = 3 bioprinted tubes per condition. **h**, Bioprinted mouse intestinal organoid pairs printed with 250-, 500- or 1,000-μm centre-to-centre organoid spacing. Tissues printed close together (~75 μm edge-to-edge) fuse (1), whereas tissues printed far enough apart (>~300 μm edge-to-edge) do not fuse (2, 3). Scale bars, 500 μm. **i**, Organoids printed in arrays with 500-μm pitch lack crypts between day 6 and 11. Arrowheads indicate crypts that formed close to the neighbouring organoids that are gone by day 11. Scale bars, 500 μm. **j**, Maximum-intensity projections of intestinal organoid arrays bioprinted at different depths within MAGIC matrix (500 μm, 1,000 μm, or 1,500 μm from the coverglass). Scale bars, 500 μm. **k**, Organoids printed deeper in the matrix (1) do not significantly grow between days 5 and 7 post-print compared with organoids printed closer to the media interface (2, 3). Data shown are mean ± s.d. of *n* = 12 organoids per condition; statistical significance was determined by non-parametric *t*-test between day 5 and day 7; values shown for *p* < 0.05; NS, not significant. Print plate illustration in **a** created in BioRender; Graham, A. https://biorender.com/64hyq2k (2026).

Organoid morphogenesis can be highly uniform when carefully controlling for initial conditions—for example, by starting from cell aggregates of similar initial size, geometry and composition^17,27,42,43^. We therefore focused on developing bioprinting modalities that use MAGIC matrices together with pelleted single-cell bioinks at tissue-like densities of ≥10^8^ cells ml^−1^ (ref. 44). We designed a piezoelectric printhead with fast pressure ramps that could directly aspirate small volumes with high precision and without excess loss due to tubing and fluidics (that is, ‘dead volume’) (Fig. 3a and Supplementary Information, section 1). We leveraged a rapid voltage switch to ‘break’ cell slurry from the printhead by applying a small negative pressure and *xz* displacement (Fig. 3b,c). We then applied this aspiration, extrusion and tail-breaking programme to script an automated spheroid bioprinting array. We first demonstrated that a Caco-2 cell slurry bioink could be delivered through a 125-μm inside diameter (ID) nozzle to create spheroids of customizable dimensions (Fig. 3d). Spheroid area was a linear function of extrusion step (Fig. 3e). After bioprinting and self-organization in MAGIC matrices, Caco-2 spheroids underwent efficient lumenization (Fig. 3d). Although the initial geometry of extruded cell volume sometimes deviated from spherical, the high active surface tensions of these living inks rapidly correct surface irregularities through self-organization (Fig. 3f).

Cell slurries could also be printed as cylinders whose diameter could be tuned using both extrusion rate and stage speed, providing multiple engineering controls (Fig. 3g). Tube diameter was an approximately linear function of extrusion rate for the two stage speeds tested. Bioprinted Caco-2 cylinders self-organized similarly to spheres while maintaining their high aspect ratio (Fig. 3g, inset). These results demonstrate that a printhead using a piezoelectric actuator affords high volume precision and flexible control over bioink behaviour.

We explored the generality of bioprinting into MAGIC matrices by preparing mouse small intestine organoid arrays (Supplementary Movie 2). An important contributor to tissue and organoid heterogeneity in traditional ECM dome cultures is the disparity in microenvironmental conditions such as media access or interorganoid spacing. We therefore systematically varied either interorganoid spacing or *z* depth within the MAGIC matrix support bath using organoids printed with an initial average diameter of200 μm. Organoid pairs printed with 250-μm centroid-to-centroid spacing came into contact as they grew and fused over time, leading to one large organoid (Fig. 3h). Organoids spaced 500 or 1,000 μm apart remained distinct and appeared to undergo normal morphogenesis for at least 7 days of culture. To examine the effect of interorganoid spacing on morphogenesis we printed 3 × 3 arrays with 500-μm spacing. Notably, after 11 days in culture, crypts only appeared at the periphery ofthe organoid array, while the centre organoid lost crypts (Fig. 3i). We hypothesize that this could be caused either by nutrient depletion or autocrine gradients^45^. Printing depth also had a profound effect on organoid phenotype, with organoids printed closer to the ECM–media interface growing the largest and forming the most crypts (Fig. 3j). Organoids printed too far from the ECM–media interface did not significantly grow over the same time (Fig. 3k). Together, these results highlight the importance ofinitial conditions and local microenvironment on organoid morphogenesis.

### MAGIC matrix enables the self-organization of many organoid types following bioprinting

**Extended Data Fig. 5 |:**
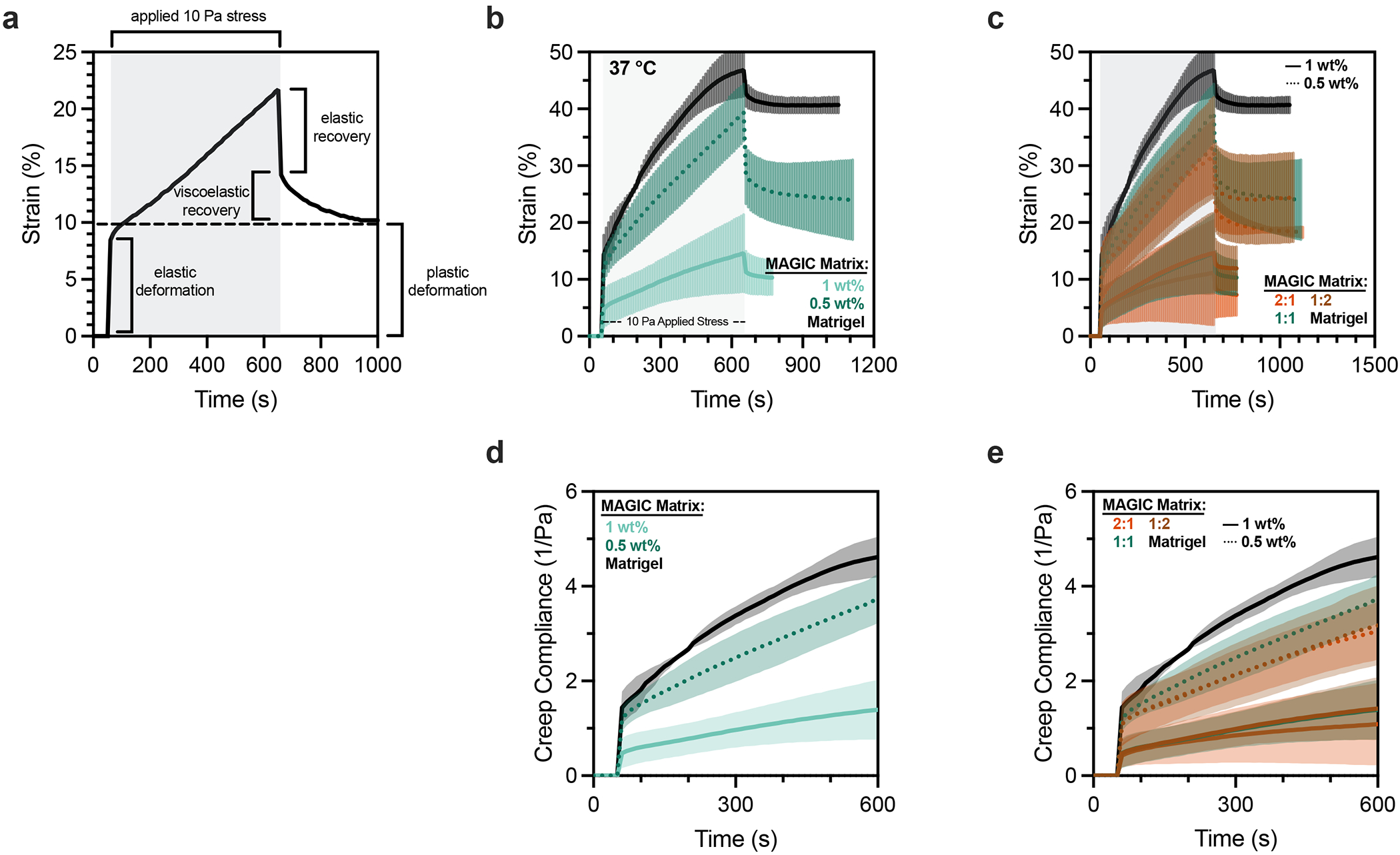
MAGIC matrices promote canonical self-organization of various organoid types from different developmental lineages. **a**. Bright-field and fluorescent live images of mouse intestinal organoid arrays 2 days after bioprinting expressing either mTomato or *Lgr5*-eGFP. eGFP signal localizes to the base of the crypts, where *Lgr5*+ stem cells should reside. Scale bars = 500 μm. **b**. Left, maximum intensity projections of DAPI, *Lgr5*-GFP, ECAD-stained intestinal organoid arrays 3 days after bioprinting. Scale bar = 1 mm. Right, 3D rendering of fixed bioprinted intestinal organoid stained for DAPI, ECAD, and Paneth cells (LYZ). Scale bar = 200 μm. **c**. Maximum intensity projection of DAPI, ECAD, and enteroendocrine cells (CHGA). Crypts protrude in all directions, highlighting fully 3D morphogenesis in MAGIC matrices. Scale bar = 200 μm. **d**. Live imaging of bioprinted salivary gland organoid arrays expressing mTomato. Scale bar = 1 mm. **e**. Immunofluorescence of fixed bioprinted salivary gland organoids showing presence of both basal (keratin 14) and ductal (keratin 8) cells. Scale bar = 100 μm. **f**. Bright-field images of HUVEC cords bioprinted at different stage translation speeds highlighting control over vessel width. Scale bars = 1 mm. **g**. 3D rendering of an AF647-NHS stained MAGIC matrix tube printed into an AMG bath, highlighting the ability to pattern ECM. **h**. Bright-field images of mouse embryonic fibroblasts printed as two nodes with patterned Collagen 1 and MAGIC matrix printed between the nodes. Fibroblasts spread only into patterned area, highlighting spatial control over cell migration. Scale bar = 500 μm.

**Fig. 4 |:**
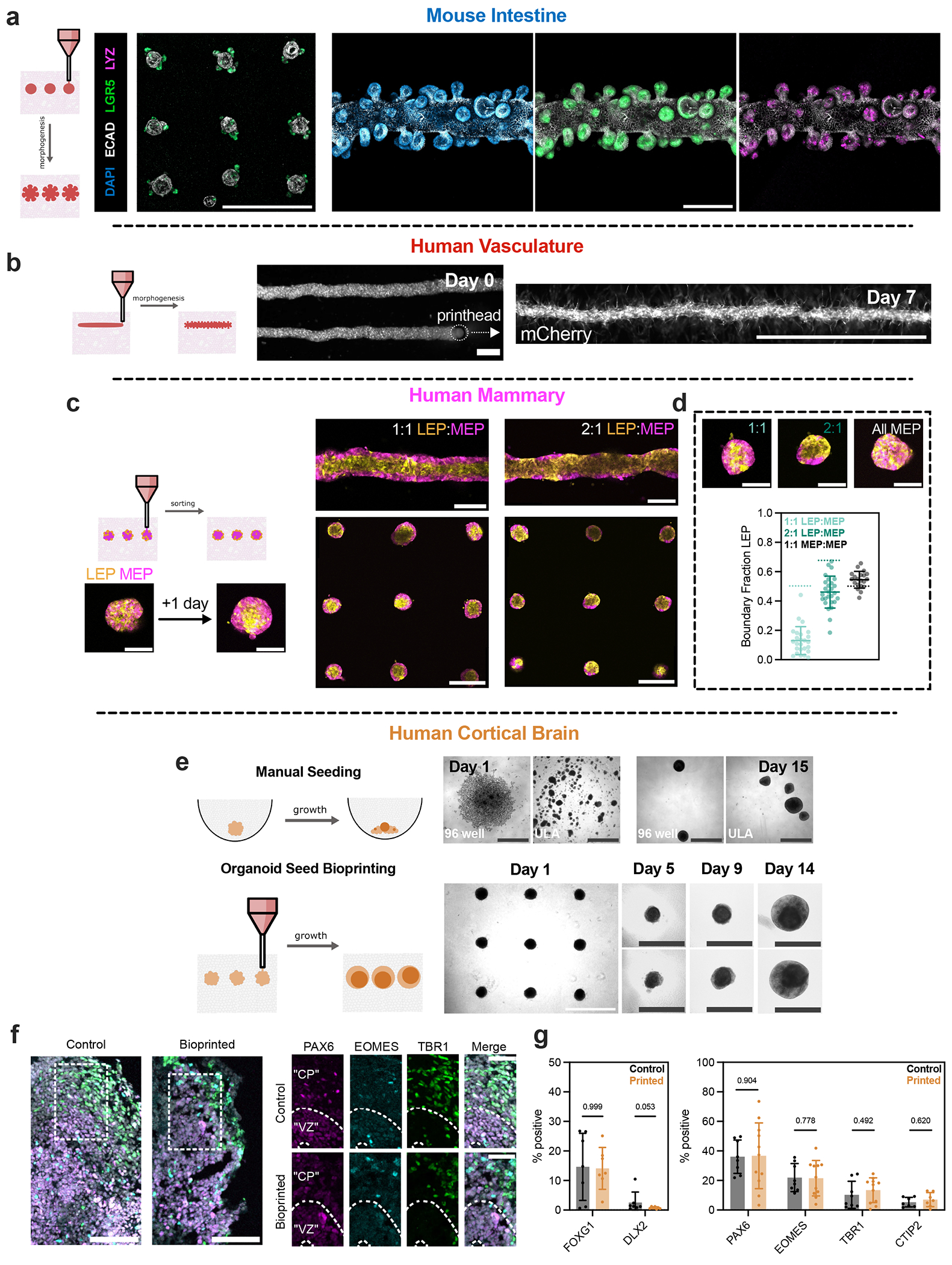
MAGIC matrices promote self-organization of bioprinted tissues derived from the three primary germ layers. **a**, Left: maximum-intensity projections of a representative *Lgr5*-GFP- and ECAD-stained intestinal organoid array 3 days after bioprinting. Scale bar, 1 mm. Right: maximum intensity projections 3 days after bioprinting intestinal organoid tubes stained with of DAPI (left), *Lgr5*-GFP (middle) and LYZ (right). Scale bar, 200 μm. **b**, Representative live images of bioprinted mCherry-expressing HUVEC tubes during printing (day 0) and following self-organization (day 7). Tubes over 2 mm long could be printed, with signs of vascular sprouting. Scale bars, 200 μm (day 0) and 1 mm (day 7). **c**, Maximum-intensity projections of bioprinted spheroid arrays and tubes of HMEC organoids of different luminal and myoepithelial compositions. Organoids were allowed to sort for 1 day following printing. Scale bars, 500 μm (arrays and tubes), 200 μm (individual spheroids). **d**. Representative live images and quantification of luminal cell boundary occupation in bioprinted organoids as a function of composition. Dashed lines represent expected boundary occupancy based on starting composition for mechanically equivalent cells^43^. Data shown are mean ± s.d. for *n* ≥ 20 organoids analysed per composition. Scale bars, 200 μm. **e**, Comparison of manually seeded and bioprinted induced pluripotent stem cell-derived human cortical brain organoids. Bright-field images of manually seeded cortical brain organoids in 96 or ultralow-attachment (ULA) well plates (top) or bioprinted arrays (bottom) over time. Scale bars, 1 mm (array), 200 μm (manually seeded or individual bioprinted organoids). **f**, Left: 18-μm maximum-intensity projections of control or bioprinted cortical organoid sections stained for cortical identity and neuronal differentiation. Scale bars, 100 μm. Right: staining for cortical cell types. CP, cortical plate; VZ, ventricular zone. Scale bars, 50 μm. **g**, Quantification of cortical identity (left) and neuronal differentiation (right) between bioprinted and manually seeded cortical brain organoids. Data shown are mean ± s.e.m. of one quantified cryosection of *n* ≥ 3 organoids per marker from two separate differentiations; numbers shown are *p* values of multiple Mann–Whitney two-tailed tests.

We next assessed whether organoid arrays exhibited expected self-organization when printed and cultured in MAGIC matrices, including mechanisms such as lumen formation, folding, budding, sprouting and sorting. For intestinal organoids, live imaging of a tdTomato reporter revealed crypt budding and protrusion within 2–3 days after printing in 96-well microplates (Extended Data Fig. 5). Organoids exhibited characteristic *Lgr5*+ stem cell organization at the crypt base (Fig. 4a) and were positive for mature epithelial cell types, including Paneth cells (lysozyme) and enteroendocrine cells (chromogranin-A). Cell slurries derived from intestinal organoids also self-organized efficiently when printed in a cylindrical format^17,42,46^. Printed cords compacted, lumenized and folded radially, extending crypts within 2–3 days after printing.

Organoids of multiple developmental lineages that form through different programmes of self-organization behaved as expected following MAGIC matrix bioprinting. For example, mouse submandibular salivary gland, an ectodermal tissue that undergoes budding morphogenesis^47^, exhibited characteristic multilobular structures within 3 days of printing and expressed ductal keratin 8 and basal keratin 14 (Extended Data Fig. 5). Human umbilical vein endothelial cell (HUVEC) vascular cords sprouted microvessels when printed into MAGIC matrix compositions supplemented with 1 mg ml^−1^ collagen I^46^ (Fig. 4b and Supplementary Movie 3). Vessels could be tuned in length and width up to and beyond the centimetre-scale (Supplementary Movie 4). We also evaluated the efficiency of cell sorting by printing spheroid arrays of patient-derived human mammary epithelial cells (HMEC) organoids, which comprise both the luminal cell (LEP) and myoepithelial cell (MEP) lineages^43^ (Fig. 4c). Printed tissues robustly sorted across ~100-μm distances to form bilamellar structures within 1 day, with the continuity of MEP coverage in the outer layer proportional to MEP composition in the organoid (Fig. 4d). HMECs printed as tubes also sorted robustly and maintained their initial tissue tubular geometry. Thus, MAGIC matrix bioprinting is compatible with a variety of self-organization mechanisms, including sorting, lumenization, migration and tissue folding.

**Extended Data Fig. 6 |:**
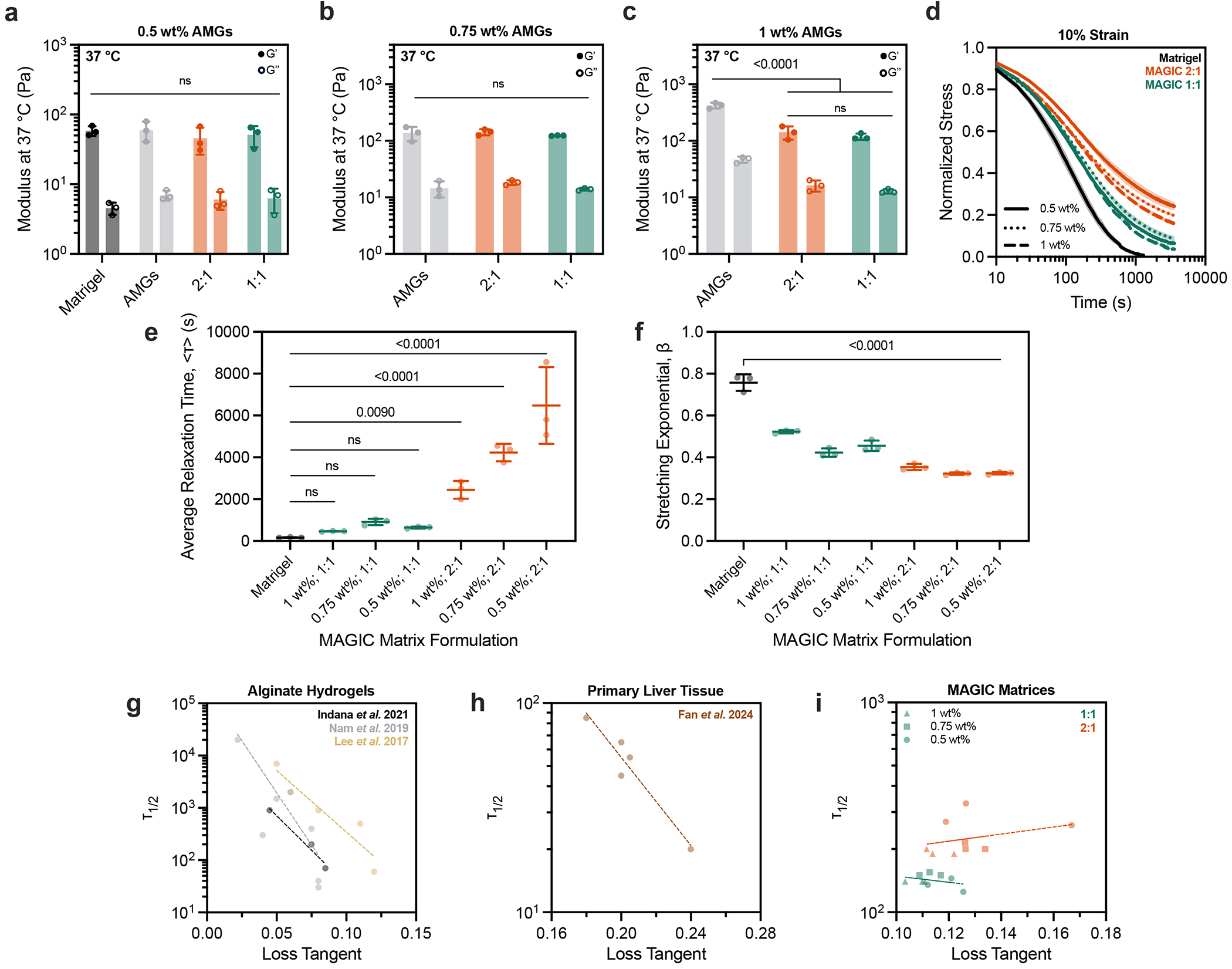
Bioprinted hiPSC-derived cortical brain organoids exhibit matrix-dependent phenotypes and rosette organization. **a**. Live images of human iPSC-derived cortical organoids from three different donors (methods) bioprinted into arrays using AMG support baths. Scale bar = 1 mm. **b**. Cortical organoids bioprinted in MAGIC matrices show sprouting (arrow) and neuroepithelial budding (arrowheads). Scale bars = 1 mm (left) and 500 μm (right). **c, d**. 20 μm maximum intensity projections of bioprinted cortical organoids stained for (**c**) cortical identity and (**d**) neuronal differentiation. Scale bars = 50 μm.

**Fig. 5 |:**
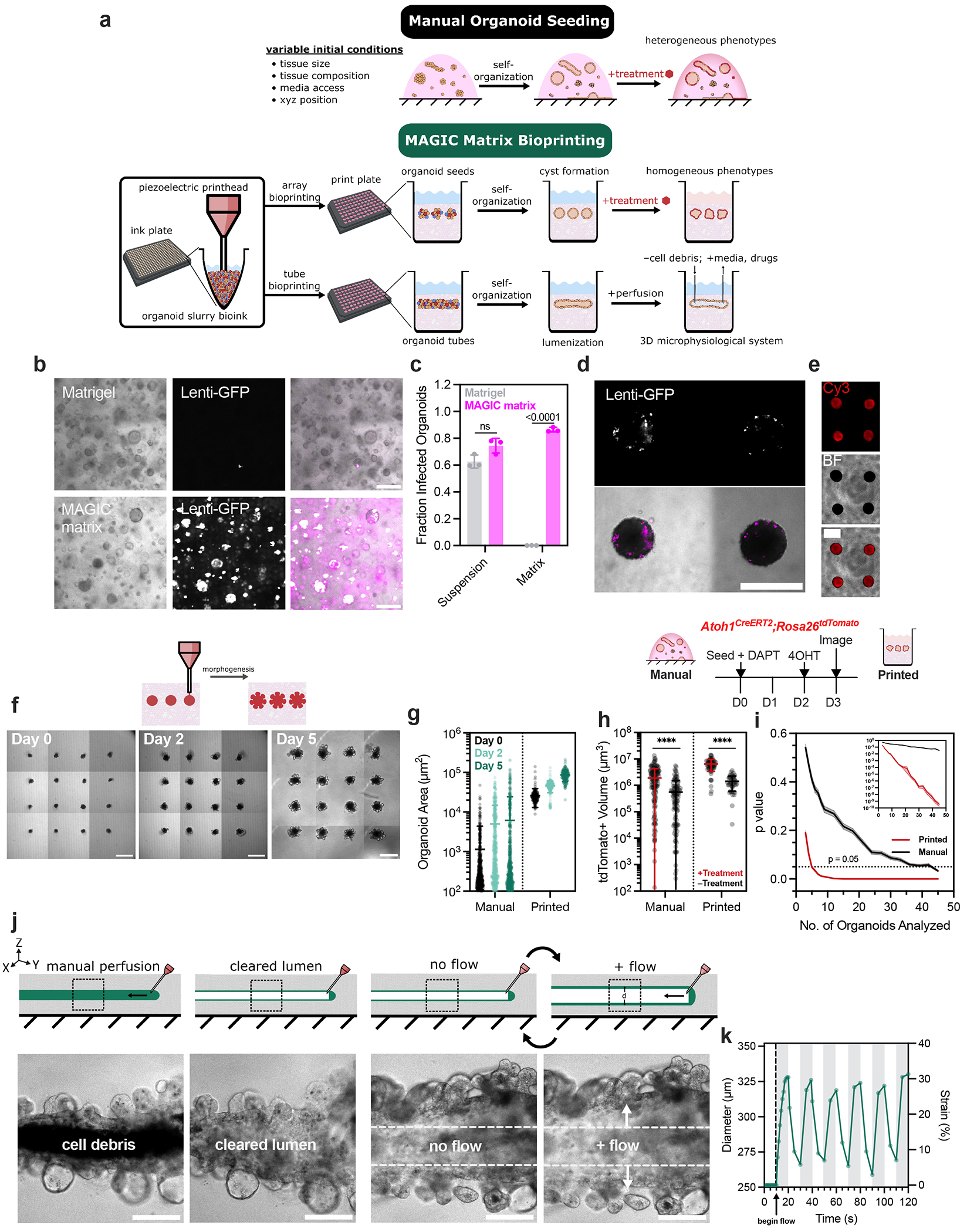
MAGIC matrix bioprinting enables generation of high-throughput organoid arrays with improved statistical power and perfusable 3D microphysiological systems. **a**, Traditional manual methods of seeding organoids lead to heterogeneity in organoid growth and morphogenesis due to heterogeneity in starting tissue size, composition and microenvironment. By controlling for initial conditions such as cell number, media access and organoid spacing, bioprinting platforms facilitate rapid generation of reproducible organoid arrays or freeform 3D microphysiological systems. **b**, Live images of TNBC patient-derived organoids transduced overnight with GFP-expressing lentivirus while seeded in Matrigel (top) or MAGIC matrix (bottom). Scale bars, 200 μm. **c**, Fraction of GFP+ organoids transduced in suspension before seeding or transduced after seeding for either ECM composition. Data shown are mean ± s.d. of *n* = 3 replicate ECM conditions; NS, not significant; ****p < 0.001 determined by two-tailed non-parametric *t*-test between ECM conditions. **d,e**, Live images of bioprinted TNBC organoids transduced with GFP-expressing lentivirus overnight after printing (**d**) or transfected using Lipofectamine and Cy3-conjugated single-stranded non-coding small RNA for 24 h, 3 days after printing (**e**). Scale bars, 500 μm. **f**, Live imaging of bioprinted intestinal organoid arrays following printing and after 2 and 5 days in culture. Scale bars, 500 μm. **g**, Organoid area over time measured by segmentation for manual or bioprinted intestinal organoids; data shown are mean ± s.d. of *n* ≥ 190 organoids per time point. **h**, Top, experimental outline of phenotypic assay for inhibition of gamma-secretase. Red fluorescence indicates *Atoh1*+ secretory progenitors. Bottom, total red fluorescence volume per organoid in treated and untreated conditions from live images of bioprinted and manually seeded organoids treated with and without gamma-secretase inhibitor. For bioprinted arrays, data shown are mean ± s.d. of *n* = 45 organoids per condition. For manually seeded organoids, data shown are mean ± s.d. of *n* ≥ 135 organoids per condition. ****p < 0.0001 determined by two-tailed non-parametric *t*-test. **i**, Bootstrapping analysis of statistical significance between treated and untreated conditions for either bioprinted or manually seeded organoids as a function of number of paired comparisons in a two-tailed non-parametric *t*-test. Curves shown are mean ± s.e.m. for *n* = 512 independent analyses. Inset: statistical significance approaches zero (<10^−9^) for bioprinted organoids using an equivalent number of comparisons because it takes manually seeded organoids to approach *p* = 0.05. **j**, Bright-field images of bioprinted intestinal organoid tubes that are manually perfused with a glass capillary attached to a micromanipulator and flushed to remove cell debris and access the lumen. Scale bars, 200 μm. **k**, Quantification of tube diameter and resulting strain upon application and removal of fluid flow. Grey bars correspond to times when fluid flow was applied. Print plate illustrations in **a** created in BioRender; Graham, A. https://biorender.com/64hyq2k (2026).

The most common approach for defining the initial conditions of organoid culture is to aggregate dissociated tissue or stem cells using low-attachment plates^1^. However, it is unclear how aggregation dynamics impact downstream morphogenesis (Fig. 4e). We reasoned that MAGIC matrix bioprinting could enhance aggregate formation by forcing cells to interact in a common volume, and tested this by bioprinting human induced pluripotent stem cell (iPSC)-derived forebrain organoids^48^. We generated a bioink from 7-week differentiated organoids and printed into both MAGIC matrix or pure AMGs. Cortical brain organoids printed into MAGIC matrix exhibited the sprouting behaviour that is expected in rBM gels, and neuroepithelial bud formation, but lacked neuroectoderm (Extended Data Fig. 6)^49^. In contrast, cortical brain organoids printed in pure AMG slurry formed dense spheroids without protrusions and exhibited spatially organized neuroectoderm (Fig. 4e). These organoids were positive for the dorsal forebrain marker *FOXG1* and negative for the ventral forebrain marker *DLX2*, with ~15% dorsal identity and <1% ventral identity (Fig. 5f,g). Furthermore, the bioprinted cortical organoids demonstrated characteristic rosette self-organization with neural progenitors surrounding ventricular zone-like regions (PAX6+, ~35%), intermediate progenitor cells (EOMES+, ~20%) and deep layer excitatory neurons (TBR1+, ~10%; CTIP2+, ~5%) extending radially out from the apical surface (Fig. 5f). Overall, bioprinted organoids showed similar cell proportions to microwell-aggregated cortical organoids. Together, these results validate organoid bioprinting for efficient aggregate formation of iPSC-derived tissues.

### Generation of high-throughput bioprinted organoid arrays for assay development

**Extended Data Fig. 7 |:**
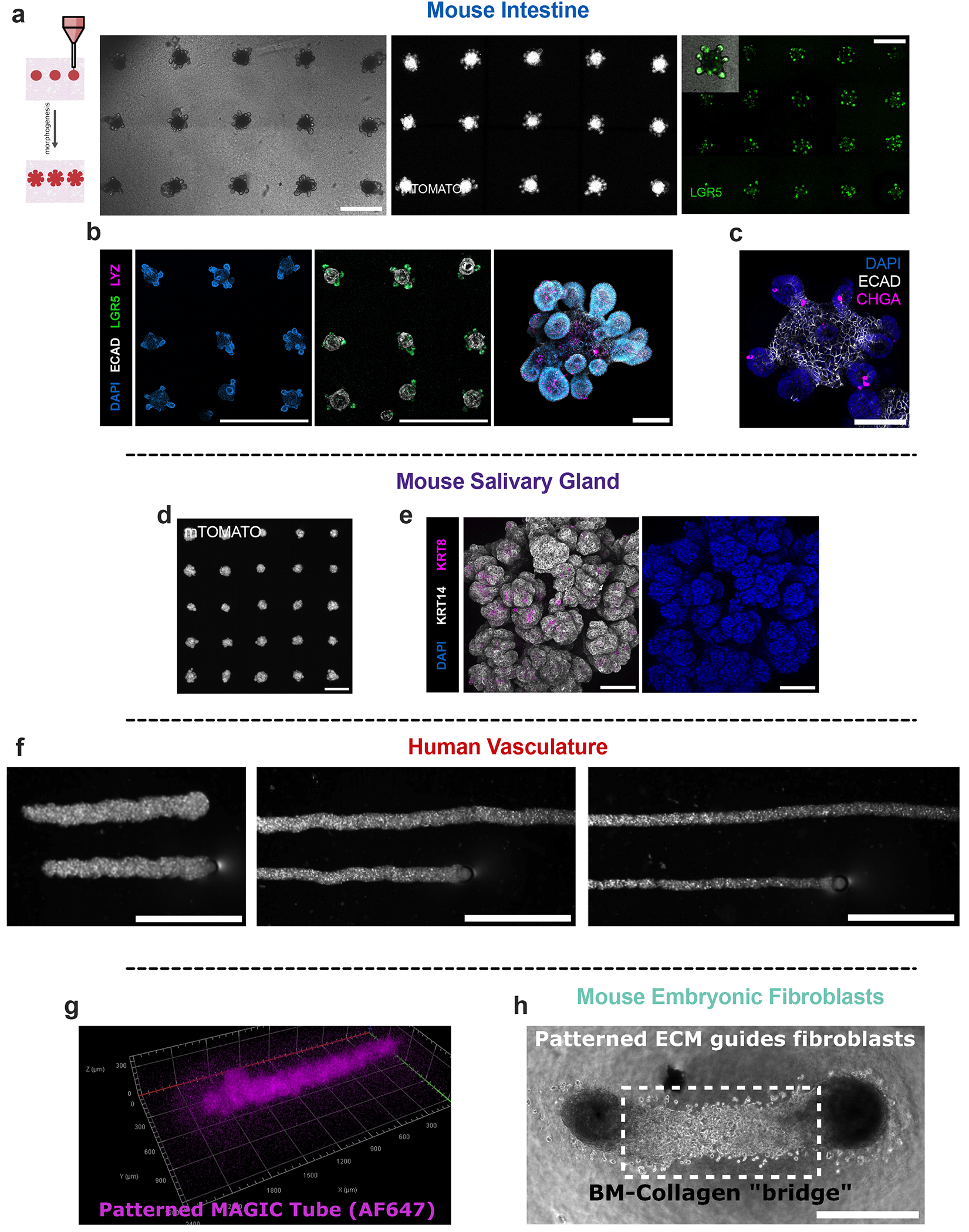
Bioprinted organoid arrays exhibit improved homogeneity and morphogenesis. **a**. Organoid area and **b**. circularity measured using max intensity projections of confocal z-stack images of GFP-expressing Caco-2 cell slurries immediately after seeding in microwells or bioprinting. Data shown are mean ± SEM of n ≥ 9 organoids per condition. **c**. Quantification of crypts per organoid for those manually seeded in Matrigel or MAGIC matrix, or bioprinted. Data shown are mean ± SD of n ≥ 30 organoids; ns = not significant; **** = p < 0.0001 determined by one-way ANOVA with Dunnett’s multiple comparisons. **d**. Picture of large (n = 528) organoid array bioprinted into a chambered slide using MAGIC matrix. Scale bar = 10 mm. **e**. Live images of bioprinted TNBC organoid arrays transfected using Lipofectamine RNAiMAX and a Cy3-conjugated, single-stranded non-coding small RNA (36mer) at various concentrations and transfection times. Scale bar = 500 μm. **f**. Live images of manually seeded and bioprinted *Atoh1*-tdTomato organoid arrays using two different DAPT treatment timelines. The left treatment timeline and images were used for bootstrapping and statistical analysis.

Similar to microwell methods, bioprinted organoid arrays hold great promise to increase the reproducibility and sensitivity of high-throughput assay development using CRISPR and drug screens^16^. However, bioprinted arrays have the added benefit of precise control over tissue geometry and other initial conditions (Fig. 5a). We compared organoid arrays prepared using MAGIC matrix bioprinting to traditional microwell aggregation. Bioprinted arrays had decreased variance in initial tissue size and improved circularity compared with microwells (Extended Data Fig. 7). To explore assays requiring genetic perturbations, we also transduced triple-negative breast cancer (TNBC) patient-derived organoids using H2B-GFP lentivirus (Fig. 5b). TNBC organoids showed no difference in infection efficiency when transduced in suspension and then plated in Matrigel or MAGIC matrix domes (Fig. 5c). However, when transducing TNBC organoids through the ECM by including lentivirus in the media after plating and gel cross-linking, infection efficiency was dramatically increased in MAGIC matrix, with nearly 90% GFP+ organoids compared to minimal-to-no infection in Matrigel. We hypothesize this is due to improved diffusivity or low protein binding afforded by low wt% (and therefore large pore size) AMGs in the composite support. Arrayed TNBC organoids were successfully transduced by including lentivirus in the media after bioprinting (Fig. 5d). TNBC organoid arrays were also highly amenable to transfection using Lipofectamine, showing strong RNA uptake as a function of both transfection time and amount of RNA delivered (Fig. 5e and Extended Data Fig. 7).

Achieving regularity among organoids with complex morphologies is a key challenge in developing organoid-based assays because it impacts the response of the organoids to genetic, mechanical and chemical perturbations^16^. We therefore quantified the timing and regularity of crypt morphogenesis after bioprinting in MAGIC matrix. Over the course of 5 days, bioprinted organoids synchronously self-organized, initially into lumenized cysts that further underwent budding morphogenesis (Fig. 5f). By contrast, manually seeded organoids showed far more heterogeneous morphologies over the same timeframe (Fig. 5g). Furthermore, bioprinted organoid arrays underwent more extensive and uniform morphogenesis, exhibiting a greater number of crypts with decreased variance compared with manually seeded organoids^50^. Organoids grown in manually seeded cultures using MAGIC matrix or pure Matrigel showed no difference in crypt number, indicating that more uniform initial conditions specifically contribute to improved maturity.

### Bioprinted organoid arrays dramatically improve assay statistical power

The uniformity and reproducibility of MAGIC matrix bioprinting could dramatically improve the sensitivity of phenotypic assays. We tested this idea by treating intestinal organoids with a gamma-secretase inhibitor, *N*-[*N*-(3,5-difluorophenacetyl)-L-alanyl]-*S*-phenylglycine *t*-butyl ester (DAPT), which is known to increase the number of *Atoh1*+ secretory progenitors and decrease the number of stem cells^51^. Using an *Atoh1^CreERT2^:Rosa26^tdTomato^* reporter line, we treated bioprinted and manually seeded organoid cultures with DAPT for 2 or 3 days after seeding and imaged after 4 or 5 days (Extended Data Fig. 7). In manually seeded organoids, differences in overall tdTomato signal acquired by confocal microscopy were obscured by heterogeneity in organoid position, size and shape. In contrast, there was a clear change in morphology and increase in signal for DAPT-treated organoids in bioprinted arrays. We hypothesize this morphological change is linked to impacts of Notch inhibition on intestinal stem cell proliferation, which may be more obvious in bioprinted organoid arrays^51^.

Quantifying the volume of tdTomato-positive signal in each condition revealed that although there was a statistically significant increase in signal for both bioprinted and manually seeded organoids in treated versus untreated conditions, the effect size was substantially improved using bioprinted arrays (~3.7-fold increased difference between treated and untreated means) (Fig. 5h). Signal readout was also not normally distributed when manually seeded (*p* < 0.0001 as determined by D’Agostino and Pearson test). Bioprinted organoids, in contrast, were more normally distributed and had decreased variance (printed coefficient of variance = 48% for treated and 58% for untreated; manual coefficient of variance = 127% for treated and 174% for untreated). Computing a post hoc power analysis (*α* = 0.05; *β* = 0.2) with the given effect sizes and variances in each condition recommended *n* = 12 printed organoids compared to *n* = 100 manually seeded organoids. This nearly order-of-magnitude decrease in required comparisons emphasizes the attractiveness of MAGIC matrix bioprinting for rare tissues or subtle phenotypes.

To more precisely quantify how bioprinted arrays improved assay sensitivity, we performed bootstrapping on the bioprinted and manually seeded populations to calculate *p*-value as a function of the number of paired organoid comparisons (treated versus untreated). In the bioprinted arrays, we observed *p*-values below 0.05 after comparing only five organoids; in manually seeded organoid cultures, 45 comparisons were required to reach the same statistical significance (Fig. 5i). *P*-values for bioprinted organoids continued to decrease with additional comparisons approaching *p* ≈ 10^−10^ after 45 comparisons—the same number of organoids necessary to reach a *p*-value of 0.05 in manually seeded organoids. This 10^8^-fold improvement highlights the potential of these bioprinted organoid arrays to detect subtle phenotypes in chemical, microenvironmental and genetic screens.

### Flexible production of 3D perfusable organoid tubes

Given the importance of fluid flow and both static and cyclic stress during processes such as peristalsis^52^, we examined whether mouse intestinal organoid tubes could be perfused following their lumenization and crypt morphogenesis (Fig. 5j). Using glass capillaries, we pierced the tissue lumen and introduced fluid using a syringe to clear internal cellular debris. By applying oscillatory fluid flow we could simulate cyclic expansion and contraction as might be experienced during peristalsis (Fig. 5k and Supplementary Movie 5). We observed radial expansion over ~30% of the perfused tubes at peak pressure enabled by the high deformability of MAGIC matrices, and unlike cultures using materials with non-physiological stiffness such as polydimethylsiloxane. Thus, MAGIC matrix tube bioprinting enables access to the organoid lumen and the introduction of biophysical cues such as fluid shear and both static and cyclic stretch, key features of microphysiological systems, while maintaining a free basal surface that ultimately can be interfaced with other tissue types.

## Discussion

Promoting reproducible and complex organoid morphogenesis requires biomaterials that mimic the dynamic boundaries encountered by tissue in vivo while also supporting engineering controls necessary for setting the initial conditions of culture. Here we developed and characterized a simple granular biomaterial that achieves both goals. MAGIC matrices are relatively simple in design, using off-the-shelf and well-characterized constituents. They are highly tunable and revealed unique relationships between the loss tangent at low strains and short timescales, and stress relaxation at higher strains and longer timescales. This was true for a variety of MAGIC matrix compositions, presenting a suite of 3D granular biomaterials with which to investigate rheological determinants of tissue behaviour. Previous studies have demonstrated that rheological properties such as storage modulus, loss modulus and stress relaxation are collectively important for symmetry breaking and morphogenesis in intestinal organoids but could not indicate whether stress relaxation was independently important. We revealed the critical importance of stress relaxation at longer timescales and larger strains, consistent with the large tissue deformations required during intestinal crypt morphogenesis. Our work and others further suggest that rBMs behave more like viscoelastic liquids over the length scales and timescales of self-organization, and this capacity to dissipate stress may be a key driver of morphogenesis^9,53^. To take advantage of these materials in preparing more uniform and complex tissues in vitro, we developed a piezoelectric printhead that precisely aspirates and extrudes cell slurries at tissue-like densities directly into MAGIC matrices, thereby allowing tissues to autonomously self-organize while minimizing variability.

Most synthetic ECMs have been designed for a particular organoid or tissue type. MAGIC matrices successfully promote the self-organization of mouse and human tissues from all major germ layers, including endoderm (intestinal), ectoderm (brain, mammary, salivary gland) and mesoderm (vasculature, fibroblasts). Focusing on intestinal organoids, we found remarkably increased homogeneity in structure and phenotype by a number of metrics, including growth, morphogenesis and maturation rate. This homogeneity yielded organoid arrays that were ‘assay-ready’ in only 2–3 days, compared to previous studies that produced organoids of similar homogeneity but required 5 days or more in culture^16,54^. In addition to intestinal organoids, all tissue types could be bioprinted and cultured in high-throughput arrays or as tubes with little-to-no changes in printing parameters or matrix composition, for example, including collagen or excluding Matrigel. By a variety of functional metrics, these organoids were indistinguishable from those cultured in pure Matrigel, suggesting that the unique mechanisms contributing to tissue folding, tissue budding and cell sorting necessary for the self-organization ofthese other tissue types are also supported in MAGIC matrix^6,43,55^. MAGIC matrix bioprinting resulted in dramatic improvements in assay statistical power, decreasing the predicted number of tissues required to identify a phenotype by nearly an order of magnitude or more compared with manual culture. Therefore, this platform presents opportunities to work with precious tissue sources such as primary patient biopsies in applications such as clinical drug screens.

Finally, these technologies provide a route to more complex and in vivo-like 3D microphysiological systems, for example by perfusing bioprinted organoid tubes. The regularity and scalability of these models could be combined with established perfusion and coculture methods without the drawbacks of artificial interfaces and geometries. These findings lay the foundation for building even more complex and reproducible models ofhuman and animal development, homeostasis and disease.

## Online content

Any methods, additional references, Nature Portfolio reporting summaries, source data, extended data, supplementary information, acknowledgements, peer review information; details of author contributions and competing interests; and statements of data and code availability are available at https://doi.org/10.1038/s41563-026-02519-4.

## Methods

### MAGIC matrix preparation

Sodium alginate (Sigma, 9005-38-3) was dissolved at 1 wt% (typically 1 g into 100 ml) into preheated 60 °C sterilized double-distilled water and stirred for 2–4 h until the solution was homogeneous. In a separate flask, calcium carbonate was mixed at 0.2 wt% (typically 200 mg into 100 ml). The dispersed calcium carbonate suspension was added to the alginate solution and cooled to room temperature while stirring for 1 h, leading to 0.5 wt% alginate and 0.1 wt% CaCO_3_. Pure acetic acid (Sigma, 64-19-7) was slowly added in dropwise, at a 1:500 ratio (typically 400 μl) under constant stirring at 1,000 r.p.m. The solution increased in viscosity, indicating release of Ca^2+^ ions and alginate cross-linking. The solution was left stirring overnight at 1,000 r.p.m. to shear the mixture, leading to microgel formation. The next day, this mixture was blended for 60 s on the ‘high’ setting using a Hamilton Beach commercial blender (BioSpec 908). The microgel mixture was then strained through a 100-μm filter to remove large particles. The filtered microgels were centrifuged at 20,000*g* for 20 min at 4 °C and the supernatant aspirated, followed by resuspension in 1× volume of DMEM:F12 (UCSF Cell Culture Facility) medium supplemented with penicillin/streptomycin, Primocin (Invivogen) and 2 mM NaOH, and stored at 4 °C overnight or until the day before use. The day before use, the mixture was again centrifuged under the same conditions and the media replaced with 2× volume of DMEM:F12 supplemented with penicillin/streptomycin, Primocin, 4% v/v HEPES (1 M, UCSF Cell Culture Facility) and 4% v/v NaHCO_3_ (37 g l^−1^ in water, pH 9.5 with NaOH). This mixture was left overnight, centrifuged the next day and the supernatant aspirated. The resulting packed microgels were considered an undiluted microgel slurry and stored at 4 °C until use.

Just before seeding or printing, the undiluted slurry was mixed in various ratios (typically 1:1 pipetted volume ratio) with liquid basement membrane (typically Growth Factor-Reduced Matrigel, Corning 354230) at 4 °C. This leads to the desired dilution of microgels in basement membrane, or MAGIC matrix, for seeding or printing. The matrix was kept cold throughout seeding or printing, and then moved to a CO_2_ incubator at 37 °C for 5–10 min after seeding or printing to allow for basement membrane cross-linking. Cell culture media specific to the bioprinted tissue type was then added.

### Rheological measurements and analysis

All rheological measurements were performed using a 25-mm cone-and-plate geometry on an Anton Parr rheometer. For shear oscillatory measurements, both frequency and amplitude sweeps were performed, with constant 1% strain and 1-Hz frequency, respectively. Temperature sweeps were performed at 1% strain and 1-Hz frequency, with 60 s for temperature equilibration before each measurement. For reversible shear experiments, 1% or 100% strain was applied at 1 Hz for 1 min each. To calculate the specific yield-stress of each composition, unidirectional shear measurements were performed at 1% strain and the data were fit to a Herschel–Bulkley power-law model. The *y* intercept was used to determine the yield-stress. For stress-relaxation experiments, the same geometry was used to apply 1%, 10% or 100% strain over 1 h after cross-linking by temperature ramp for 5 min and sealing the hydrogels with mineral oil to prevent evaporation. Nanoindentation experiments were performed using an Optics11 Life Chiaro Nanoindenter with a 50-μm tip and a 0.5 N m^−1^ cantilever on indentation mode with 5-μm indentation for 60 s.

### Intestinal organoid isolation

Intestinal organoids were generated from the duodenum of Lgr5^DTR^ mice using previously described protocols^41^. Briefly, the proximal portion of the small intestine was isolated, filleted open and rinsed 2× in PBS + penicillin/streptomycin on ice. The tissue was then incubated in harvest buffer (PBS + penicillin/streptomycin + 2 mM DTT + 10 μM Y27632 + 1 mM EDTA) with gentle rocking on ice for 15 min. After 15 min, the tissue was shaken for 1 min, transferred to crypt dissociation buffer (PBS + penicillin/streptomycin + 2 mM DTT + 10 μM Y27632 + 5 mM EDTA), and incubated for 1 h with gentle rocking on ice. The tissue was then shaken for 1 min, filtered through a 70-μm filter, rinsed with 5 ml of ice-cold basal medium (Advanced DMEM/F12 High Glucose, *N*-acetyl-L-cysteine, penicillin/streptomycin, Glutamax, HEPES) and enumerated. Approximately 1,000 crypts were plated per well in preheated 24-well plates in 50-μl Matrigel domes (356231, Corning) in ENR medium (Basal Medium, B27 Supplement, N2 Supplement, hrEGF (50 ng ml^−1^), Noggin (100 ng ml^−1^) and R-spondin conditioned medium).

### Intestinal cell and organoid culture

In general, cells or organoids were cultured according to standard protocols specific to that cell type. Caco-2 cells were cultured in DMEM + 10% FBS with media changes every 3–4 days and passaged once per week. For mouse proximal small intestinal organoids (gut organoids), organoids were cultured according to published protocols^41^. Briefly, gut organoids were cultured in 3D Matrigel domes on 24-well plates with 1 ml of ENR medium (EGF, Noggin, R-Spondin) for 4–7 days before passaging, with media changes every 2–4 days, depending on desired growth rate. For passaging, gut organoids were collected by mechanical dissociation ofthe Matrigel domes using 1 ml ofcold basal medium and centrifuging. All centrifugation steps were performed at 160*g* and 4 °C for 4 min unless stated otherwise. The supernatant was aspirated, and the pellet was fragmented by pipetting up and down in 1 ml of basal medium BM using a non-filtered 10- to 100-μl pipette tip attached to the end of a 1,000-μl pipette tip to gently increase shear. The crypt fragments were collected by again centrifuging and the supernatant was aspirated. These fragments were resuspended in Matrigel, generally at a 1:4 or 1:6 split ratio (for example, 100 or 150 μl of Matrigel per 25-μl dome), and plated onto the bottom of 24-well plates. The plate was then inverted to prevent organoid settling, and the Matrigel allowed to polymerize in a CO_2_ incubator at 37 °C for 5–10 min before adding 1 ml of warm ENR to each well. Gut organoids that were to be used for bioprinting were instead cultured in 1 ml ENRCV (with added 3 μM CHIR99021 and 1 mM valproic acid) to promote stem cell expansion. Salivary gland organoids were cultured using a similar method and medium as described above, using ENR supplemented with Y27632 (10 μM), Fgf10 (100 ng ml^−1^) and Fgf2 (25 ng ml^−1^) during maintenance and before printing. These organoids were also collected, dissociated and bioprinted using the same method as for the small intestine organoids.

### MAGIC matrix bioprinting workflow

Mouse intestinal organoids were collected for bioprinting 2–3 days after passaging, when the culture consists mostly of large clear cysts without substantial internal dead cell debris, and were prepared for single-cell dissociation as described previously^17^. For most bioprinting experiments, either 10 or 20 Matrigel domes were collected, yielding roughly 1–2 million cells. As during passaging, gut organoids were collected by mechanical dissociation of the Matrigel domes. After centrifugation, the pellet was gently resuspended in dissociation medium consisting of TrypLE Express (Gibco) supplemented with 2,000 U ml^−1^ DNase I (STEMCELL Technologies), 1 mM *N*-acetylcysteine (Sigma), and 10 μM Y27632 (R&D Systems); 1 ml of this mixture was used for every ~5 wells used for printing, so generally 2 or 4 ml. The resuspended cells were incubated for 10 min in a water bath at 37 °C, with gentle shaking to agitate the pellet every 5 min. This mixture was neutralized by adding 8 ml or 16 ml basal medium supplemented with 10% FBS and gently pipetting up and down. Using a serological pipette, the cells were extruded dropwise through a 40-μm strainer to ensure a roughly single-cell suspension and filter debris. This mixture was centrifuged and the supernatant aspirated. The pellet was resuspended in 1 ml of ENR supplemented with 2.5 μM thiazovivin (Stemgent) and 2 mM EDTA (Gibco) and centrifuged again. This pellet was resuspended in ~30 μl of of ENR with thiazovivin and EDTA and transferred to the 384-well collection plate on the bioprinter. Caco-2 cell slurries and salivary gland organoid slurries were prepared using the same protocol.

As described above, undiluted AMG slurry was mixed in a 1:1 pipetted volume ratio with liquid reconstituted basement membrane matrix (typically growth factor-reduced Matrigel, Corning 354230) at 4 °C to create MAGIC matrix. Then, 100 μl of MAGIC matrix was then deposited into each well of a chilled 96-well plate (printbed plate) using a positive-displacement pipette to minimize bubbles. The printhead was then equipped with a 75-, 125- or 200-μm ID plastic denudation micropipette (CooperSurgical, EZ-Tip) to use as the print nozzle. For most experiments, a 125-μm ID tip was used. The 384-well bioink plate was centrifuged at 100*g* for 1 min to pellet the cell slurry bioink at the bottom of the well. This slurry was loaded via direct aspiration (of ≤0.66 μl) into the printhead nozzle to reduce dead volume and minimize the cell slurry volume required for printing. The motorized stage and print dimensions were calibrated manually and print parameters set using a custom MATLAB script controlling both the microscope stage and the printhead. Most frequently, 3 × 3 or 4 × 4 arrays of ~200-μm-wide organoid spheroids were deposited with 750- or 1,000-μm interorganoid spacing.

After printing, the printbed plate was carefully moved to a 37 °C CO_2_ incubator and allowed to sit for 5–10 min to allow for basement membrane cross-linking. Then, 200 μl ofwarm ENR media with 2.5 μM thiazovivin was added to each well. Media was changed every 2–4 days for spheroids, and every 2 days for tubes.

### Piezoelectric extrusion bioprinter design and operation

The piezoelectric printhead was mounted at a fixed *xy* position on a cantilevered arm fastened to a Zaber LRQ075 stage for motorized *z* control. A Leica microscope DMI8 stage controller held the print plate and provided micrometre-scale control of the *xy* position and the resulting print shape. Microscope integration provided real-time imaging during the printing process, which allowed rapid identification and diagnosis of printing issues should they occur. The bioink plate holder was mounted to two Zaber LSQ150 stages for motorized access to each bioink stored in a 384-well sample plate. Bioinks were loaded by directly aspirating a user-specified volume from a well so that only the printed volume was loaded—this is especially beneficial for precious samples such as biopsies or cell populations with insufficient bioink volume for loading typical commercial bioprinting syringes. Aspiration and extrusion was driven by Physik Instrumente’s PI-841.10 piezo, which was mounted against a fluid-filled cavity with a polyether ether ketone diaphragm at the interface. A solenoid valve toggled the fluidic connection of the printhead cavity to a fluid reservoir, allowing the printhead to be filled, cleaned or purged, and then sealed for single-ended printing operation. This equipment results in a volume displacement resolution of ~10 pl, a maximum aspiration/extrusion volume of ~660 nl, and a maximum theoretical aspiration/extrusion rate of ~300 μl s^−1^ (the actual rate and resolution will be highly dependent on the rheological properties of the bioink). The entire process, including imaging, motion control, piezopipetting and fluidics, was controlled using MATLAB. Custom printing protocols were scripted for maximum repeatability, efficiency and iterative troubleshooting (Supplementary Movie 2).

The printbed plate was chilled during the experiment using cold, dry compressed air to prevent MAGIC matrix cross-linking during printing without obscuring or wetting the optics. The printbed plate temperature was monitored using a thermocouple and kept at 4–8 °C. The bioink plate was cooled with a closed-loop recirculating chiller held at 5 °C. Before each printing experiment, a calibration procedure was completed. First, to passivate and increase the hydrophilicity of the inner tubing, cavity and pipette tip surfaces, the system was incubated for 10 min with a treatment solution consisting of phosphate-buffered saline (PBS), 10 mg ml^−1^ bovine serum albumin (BSA), 10 mg ml^−1^ Tetronic 90R4 (Sigma 435546 functional oligomer) and 5 mM EDTA to reduce adherence of cellular components and Matrigel. This procedure also reduced adherence of air bubbles, which increase the system’s hydraulic compliance and compromise pipetting precision and responsivity. Next, the pipette tip was centred within the field of view using the manual centring screws on a Thorlabs CXY1 stage. The bioink plate wells were then centred relative to the pipette tip using a three-corner calibration scheme. Finally, with the desired ink well in place, the desired aspiration height of the printhead pipette tip was set, typically just above the bottom of the bioink well, to collect dense cell slurry.

Once the instrument was calibrated, a series of scripts executed the printing procedure according to user-specified parameters. These parameters were defined in an editable file of constants, and included specifications such as print shape and size, array formatting, and the rate and amount of bioink that was loaded. The first script prompted the user to define the print area by selecting four corners of a bounding box within the print well. The printed array or tubes were constrained by the bounding box so that the tip never collided with the well walls during printing. Next, the slurry was loaded from the ink well to the tip. The tip navigated to its calibrated loading height, then aspirated slurry according to the user-specified parameters. Next, the tip navigated to the bounding box and started printing the array or tubes. The array positions were computed according to user-specified row and column dimensions while staying within the bounding box. A small back-pressure was applied by changing the voltage applied to the piezo, immediately followed by a rapid *z*-axis offset, sometimes followed by an *xy*-axis offset, to detach the cell slurry from the tip after each bolus. In general, a negative extrusion step of 0.01 μm and a *z*-axis translation of 100 μm at 1 mm s^−1^ consistently detached the slurry with minimal disturbance to the printed bolus or tube. This process was repeated for each array element within a print well and could be programmed to repeat across multiple wells for any container geometry, including microplates, Petri dishes and chambered slides.

### Organoid size and crypt analysis

Quantification of organoid area and crypt number was performed based on binary masks constructed using Fiji/ImageJ. Masks were achieved by taking maximum *z* projections of all imaged organoids in the tdTomato+ channel, adjusting contrast, applying a Gaussian blur (5–20 μm) and adaptively thresholding. Following conversion to a mask, holes were filled and organoids were identified with Analyze Particles (excluding objects < 5,000 μm^2^, parameter = exclude add). To quantify organoid area, the area metric was extracted with ROI Manager (measure). To further quantify the number of crypts, we used a MATLAB crypt counting software developed by Montes-Olivas et al.^50^. Here, we had to filter out smaller, manually seeded organoids to maintain crypt-counting accuracy. Consequently, the organoids included in this analysis were biased toward larger organoids, of similar size to the bioprinted condition.

For microwell comparison experiments, Caco-2 cells were prepared as described above and counted using a haemocytometer to calculate the desired cells seeded per EZSphere (AG4860-900SP) microwell. Cells were centrifuged to pellet at the base of the microwells before Matrigel was added on top of the wells and allowed to solidify. Media was then added to the wells and seeded spheroids were imaged and analysed as described above. For area quantification, the median 80 detected objects per well were used from two wells per seeding condition to match the 80 microwells per well EZSphere density and to exclude imaging artefacts.

### Bioprinted tube perfusion

Bioprinted tubes were allowed to self-organize and form lumens for 3–7 days, with media changes every other day. Gut tubes were generally printed into #1.5 coverglass-bottomed chambered slides with one chamber, to allow access with a glass capillary and micromanipulator. Aluminosilicate glass micropipettes with a long taper were prepared using a P-97 micropipette puller (Sutter Instruments). The pulled pipettes were cut 3–5 mm from the tip to get 10- to 25-μm-diameter pipettes with jagged ends, and filled with PBS. Once patent lumens were visible, one end of a tube was cut with a razorblade to create an opening, and the other end was pierced with the capillary mounted on a Narishige MM-89 micromanipulator connected to a syringe. Applying pressure to the syringe induced liquid and debris flow toward the open end of the tube. Images were acquired using a 10×/0.25 numerical aperture air objective on a Zeiss Axiovert 200M running SlideBook software. Tube diameter as a function of time was measured by manual thresholding of the tube and dividing the thresholded area by the imaged tube length to obtain an average tube diameter. This was performed over each frame of a video over multiple pulse cycles.

### Gamma-secretase inhibition experiment and analysis

*Atoh1^CreERT2^:Rosa26^tdTomato^* gut organoids were isolated from proximal mouse small intestine and cultured as described above. Organoids were bioprinted or manually seeded and immediately cultured in ENR supplemented with either 50 μm DAPT (+treatment) or a DMSO vehicle control (–treatment). The media was changed on the second day, and 1 μM 4-hydroxytamoxifen (4-OHT, Sigma-Aldrich H7904) was added. On the third day after seeding, organoids were imaged using equivalent settings in high-throughput on a GE Healthcare IN Cell Analyzer 2200 confocal microscope in bright-field and 568-nm channels equipped with a 10x/0.35 numerical aperture air objective. For microscopy images, a maximum intensity projection was created in the 568-nm channel to show the tdTomato+ signal, and a representative central focal plane was chosen in bright-field. For analysis, a custom Fiji macro was generated to identify and segment individual organoids. The tdTomato+ volume of each individual organoid was then calculated in Fiji using a custom thresholding macro. Bootstrapping and subsequent *p*-value quantification between treated and untreated conditions for either printing or manual seeding was performed using a custom R script^43^ with 512 iterations at each number of pairwise organoids.

### Statistical analysis

Sample numbers for a given experiment are provided in each figure legend and were always *n* ≥ 3 independent replicates. Statistical analyses included non-parametric *t*-tests, one-way analyses ofvariance (ANOVAs) with Tukey’s or Dunnett’s multiple comparisons, normality tests, conventional power analysis using *α* = 0.05 and *β* = 0.2, and bootstrapping as described above. Statistical analysis was performed using GraphPad Prism 10 (*t*-tests, ANOVAs, normality, power analysis) or R (bootstrapping).

### Additional methods

Additional methods on the culture and characterization of intestinal, salivary gland, vascular, mammary, cortical and TNBC organoids are available in the Supplementary Information.

### Reporting summary

Further information on research design is available in the Nature Portfolio Reporting Summary linked to this article.

## Extended Data

**Extended Data Fig. 8 |:**
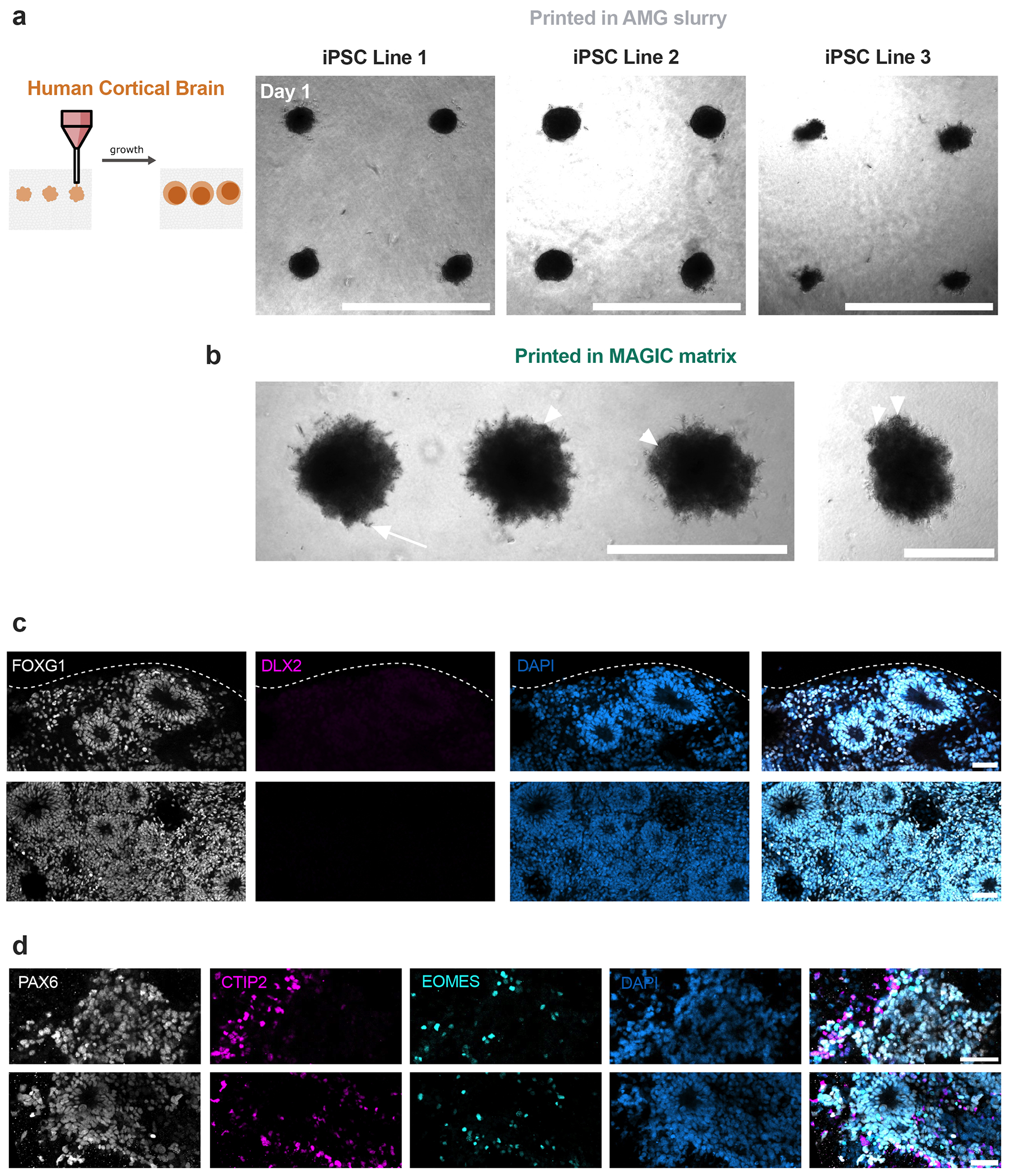


**Extended Data Fig. 9 |:**
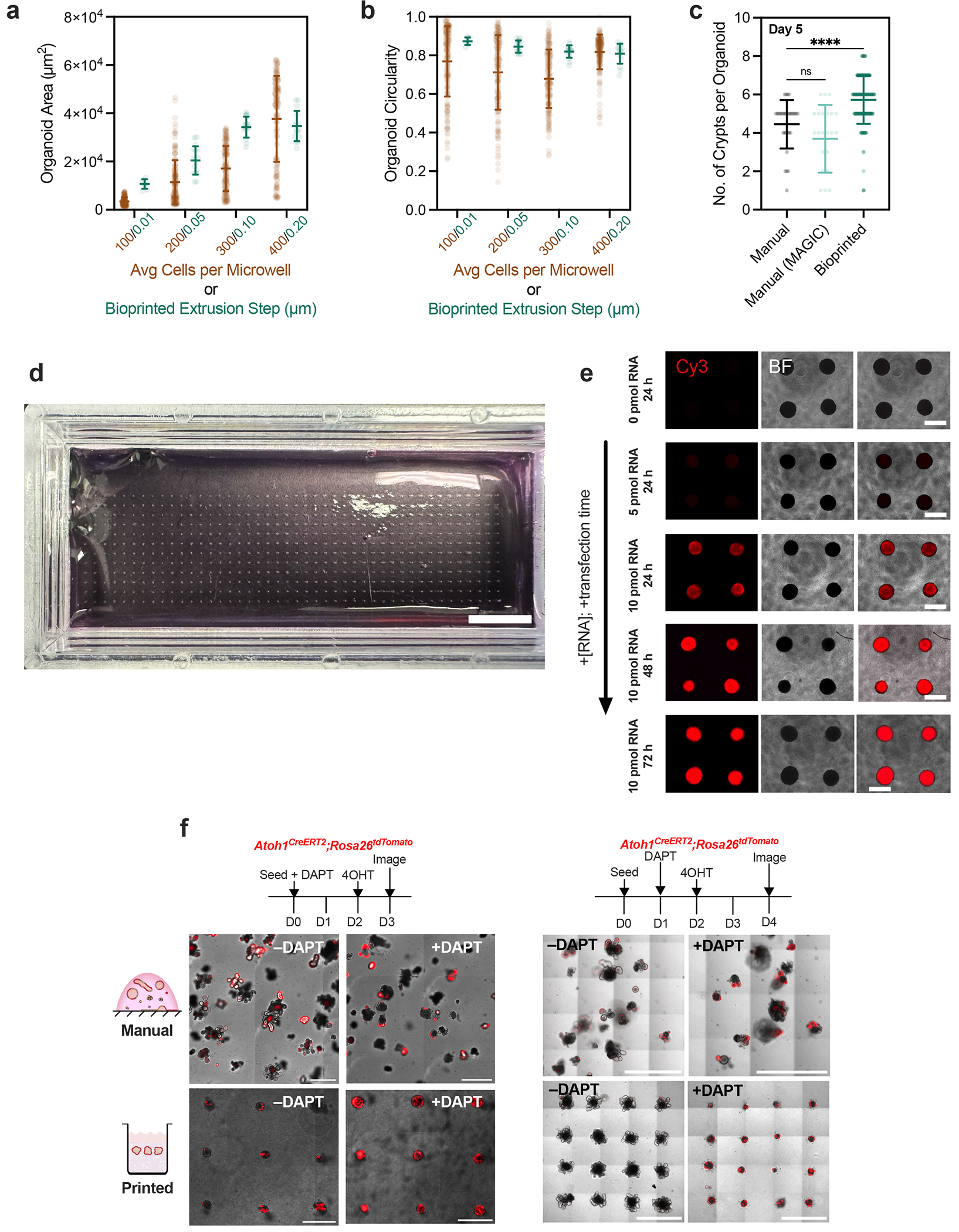


## Supplementary Material

Supplementary Video 1

Supplementary Information

Supplementary Video 2

Supplementary Video 3

Supplementary Video 4

Supplementary Video 5

The online version contains supplementary material available at https://doi.org/10.1038/s41563-026-02519-4.

## Data Availability

All source data is available at the online version of this article. Source data are provided with this paper.

## Code availability

Custom MATLAB scripts are available at Zenodo via https://doi.org/10.5281/zenodo.18331666 (ref. 56).
