# Supplementary figures and images for: Stress-relaxing granular bioprinting materials enable complex and uniform organoid self-organization
Source: Nat Mater. 2026 Mar 10;25(7):1239–51. doi: 10.1038/s41563-026-02519-4 (PMC13178435; doi:10.1038/s41563-026-02519-4)

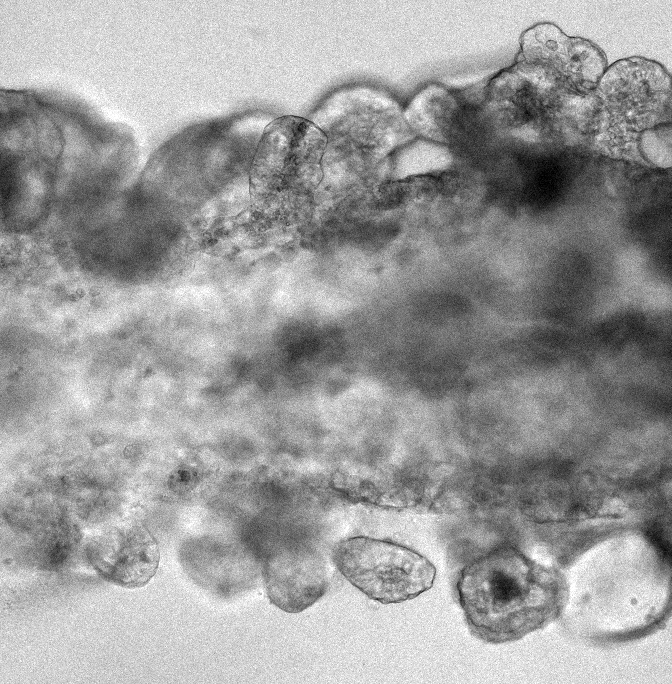

Supplement: Supplementary file 7 — Cyclic 3D pressurization of a perfused intestinal organoid tube. Tube diameter increases and decreases upon application and removal of pressure, indicating the tissue is experiencing strain. [file 41563_2026_2519_MOESM7_ESM.gif]
