# Supplementary material for: Stress-relaxing granular bioprinting materials enable complex and uniform organoid self-organization
Source: Nat Mater. Author manuscript; Available in PMC 2026 May 16. (PMC13178435; doi:10.1038/s41563-026-02519-4)
Supplement: Supplementary file 1 [file NIHMS2168536-supplement-Supplementary_Information.pdf]

Table of Contents

1. **Supplementary Results & Discussion** 2
2. **Stress Relaxation Quantification** 3
3. **Supplementary Methods** 4
4. **References** 6

1. Supplementary Results & Discussion

Independent control over modulus and stress relaxation in MAGIC matrices uncovers the contributions of ECM rheology to crypt morphogenesis

Our findings on the importance of stress dissipation in MAGIC matrices were further supported by creep-recovery measurements at 37 °C using 10 Pa applied shear stress over 10 minutes to simulate the forces and timescales of processes such as lumen expansion and crypt budding[1,2] (Supplementary Information Fig. 1). The creep response was a strong function of material composition, with Matrigel and MAGIC matrices comprising soft (0.5 wt%) AMGs exhibiting the greatest strain rate and highest plasticity.

Furthermore, to investigate whether decoupling of short and long length- and timescale stress relaxation was a general feature of MAGIC matrices, we repeated these measurements in formulations spanning three alginate wt% in the microgels and 1:1 and 2:1 volume fractions. Indeed, for all compositions, stress relaxation rate and magnitude could be tuned for materials with matched storage and loss moduli (Supplementary Information Fig. 2). While storage and loss moduli were a function of wt% in the AMG fraction, they were independent of volume fraction for 1:1 and 2:1 MAGIC matrix formulations. Furthermore, the volume fraction of AMG dictated the stress relaxation profiles independent of wt% in the microgels, with 2:1 matrices relaxing at a slower rate than 1:1. Interestingly, stiffer microgels corresponded to decreased average relaxation time. While the loss tangent and relaxation properties are often coupled for homogenous or non-granular viscoelastic materials[3–10], these data suggest that these may be de-coupled at larger deformations for granular materials with viscous interstitium, possibly due to microgel yielding and rearrangement. These results suggest that tuning microgel packing and geometry may present unique opportunities to independently investigate the roles of loss tangent and stress relaxation for small and large deformations on 3D tissue behaviors.

A piezoelectric printhead enables precise, automated direct "writing" of dense cell slurry bioinks

We designed a printhead that could directly aspirate small volumes with high precision, fast pressure ramps, and without excess loss due to tubing and fluidics (i.e. "dead volume"). We used a piezoelectric actuator that is mechanically coupled to a fluid-filled cavity via a polyether ether ketone (PEEK) diaphragm (Fig. 3a)[11]. Voltage applied to the piezoelectric material to be translated into precise expansion or contraction of the diaphragm, and therefore, aspiration and dispensing of precise volume of cell suspension. The printhead was controlled with motorized arms and mounted on a standard commercial microscope to facilitate alignment and live analysis of prints. Using this piezoelectric extrusion bioprinting setup, it was possible to print large tissue arrays comprising >200 individual organoids beginning with fewer than 10^6 cells as a saturated slurry.

High-viscosity ink droplets such as cell suspensions are challenging to print in yield-stress fluids due to inertial, cohesive, and shear forces between the ink and matrix[12,13]. For example, cohesive interactions between cells in the slurry causes the ink to be pulled as a continuous tail from the tip as it is removed from the bath, generating a structure reminiscent of capillary bridging[14,15]. To combat this, we leveraged a rapid voltage switch on the piezo printhead to "break" this tail by applying a small negative pressure and xz-displacement (Fig. 3b, c).

MAGIC matrix enables the self-organization of many organoid types following bioprinting

Using mouse embryonic fibroblasts, we demonstrated that matrix-in-matrix patterning between organoids could also control collective migration along pre-defined tracks using a 200 µm ID nozzle to print ECM (Extended Data Fig. 7).

The most common approach for defining the initial conditions of organoid culture is to aggregate dissociated tissue or stem cells using hanging droplets or low attachment plates[16]. However, spheroid formation using these methods can occur along complex or inefficient trajectories and are strongly dependent on the ability of the cells to aggregate and compact. Consequently, the process of aggregation often includes a field of unincorporated or dead cells at the periphery of the organoids. It is unclear how these complex aggregation dynamics might impact downstream morphogenesis (Fig. 4e). We reasoned that MAGIC matrix bioprinting could enhance aggregate formation by forcing cells to interact in a common volume. To test this idea, we

explored the feasibility of bioprinting human induced pluripotent stem cell (iPSC)-derived forebrain organoids[17,18].

Discussion

To demonstrate the power of MAGIC matrices, we tuned their chemical and rheological properties to match those of rBMs (such as Matrigel) while supporting embedded 3D printing. The rheological features of rBMs that support organoid growth and morphogenesis have largely been attributed to their soft, viscoelastic nature. Our work and others further suggest that rBMs behave more like viscoelastic liquids over the length- and timescales of self-organization, and this capacity to dissipate stress may be a key driver of morphogenesis[19,20]. Synthetic biomaterials incorporating appropriate viscoelasticity and some degree of stress relaxation were also shown to support 3D symmetry breaking, spreading, invasion, and differentiation[4,19,21–27]. However, in all of these studies, loss tangent is strongly correlated with stress relaxation at long timescales, making it impossible to determine how stress relaxation independently contributes to tissue morphogenesis. By modulating the volume fraction of the granular medium, we tuned stress relaxation at long timescales and high strains without affecting loss tangent, allowing us to determine the quantitative impact of stress relaxation alone on crypt morphogenesis. Our findings highlight the importance of complete dissipation of internal stresses over the larger timescales and deformations relevant to morphogenesis. While the benchmark 1:1 MAGIC matrix composition with 0.5 wt% AMGs did not completely relax over the length and timescales measured, the residual stresses (~6% or ~0.2 Pa) were significantly below those known to be exerted by tissues during growth and morphogenesis[1,2,28]. Moreover, deformations of MAGIC matrices using nanoindentation suggest that at the tens of microns length-scale relevant to cells and tissues, MAGIC matrices may relax stresses at a rate and magnitude nearly identical to Matrigel. Among the suite of biomaterials suitable for embedded 3D tissue culture, MAGIC matrices allow for significantly greater stress relaxation at large deformations and timescales[10,19,26,29–31]. The tunability of stress relaxation was generalizable for the range of MAGIC matrix compositions tested, highlighting a potentially unique property of granular materials with viscous interstitium that warrants further investigation. Notably, we did not engineer the shape of the granular medium in this study, a feature predicted to control packing density[32,33] and whose optimization may increase the range of volume fractions, and thus the degree of stress relaxation, afforded by MAGIC matrices.

MAGIC matrix bioprinting held other unique features that point to future applications in high-throughput biology. For example, we found that organoid arrays fully embedded in MAGIC matrix were amenable to transfection with mRNA and transduction with lentivirus far more efficiently than in Matrigel. Their uniform position facilitated both live and fixed imaging as well as automated analysis.

2. Stress Relaxation Quantification

To quantify the relevant time scale of stress relaxation we fit stress relaxation profiles using a stretched exponential function[10] :

$$\sigma(t) = \sigma_0 e^{-(\frac{t}{\tau_c})^{\beta}};$$

where σ describes shear stress over time, β is the stretching exponent, and τ_c is the characteristic relaxation time. Values of β for Matrigel were closer to 1, indicating that the stress response is dominated by a single relaxation mode. Values of 1:1 and 2:1 MAGIC matrix were around 0.5 and 0.3, respectively (Extended Data Fig. 3). These results suggest that the relaxation modes in MAGIC matrices are more complex as the microgel fraction increases, which appears to impact long-term tissue behavior. We additionally quantified an average relaxation time for each material as recently described[10]:

$$< \tau > = \frac{\tau_c}{\beta}\Gamma(\frac{1}{\beta}).$$

For comparison of loss tangent and stress relaxation, the stress relaxation half-life, or $\tau_{1/2}$, was calculated from stress relaxation curves or obtained from published datasets[6–9] and plotted as a function of loss tangent for each reported material formulation. Data were fit using linear regression to demonstrate negative correlation between loss tangent and stress relaxation half-life for all analyzed materials besides MAGIC matrices. Data were plotted based on material identity, including alginate-based materials, primary liver tissue, and MAGIC matrices to account for material-specific differences in half-life magnitude.

3. Supplementary Methods

3.1 Salivary gland organoid isolation: Salivary gland organoids were generated from the submandibular glands (SMGs) of adult female mice (Krt14-Cre; mTmG). SMGs were dissected out and washed in ice cold PBS. SMG tissue was minced into small pieces with surgical scissors, followed by thorough mincing with a razor blade. The resulting tissue fragments were resuspended in Disaggregation Medium consisting of Serum Free Medium (RPMI1640, 1% Pen/Strep, Sodium Pyruvate, MEM non-essential amino acids, L-glutamine and HEPES) with DNAse (100 µg/mL) and Liberase TL (125 µg/mL). Tissue was incubated with shaking for 1 hour at 37°C. At 30 and 60 minutes, tissue was triturated to promote dissociation. Disaggregated tissue was filtered through a 70 µm filter, washed with 3 mL of Basal Medium and enumerated. Approximately 10,000 tissue fragments were plated per in 50 µL Matrigel domes (356231, Corning) in ENR medium supplemented with Y27632 (10 µM), Fgf10 (100 ng/ml) and Fgf2 (25 ng/ml).

3.2 Human umbilical vein endothelial cell (HUVEC) culture and matrix preparation for bioprinting: HUVECs (Lonza) were cultured in EGM-2 and used between passages 4 and 6 as described previously[34]. mCherry-HUVECs were created by transducing cells with a pSicoR-EF1a-mCherry lentivirus. All lentiviruses were made by the UCSF Viracore. Transduced cells were sorted on a BD Aria II flow cytometer. For bioprinting experiments, confluent HUVECs were digested using TrypLE Express (Gibco) for 10 min at 37 °C. Cells were washed in D-PBS with 2 mM EDTA and filtered using a 40 µm cell strainer. Single-cell solutions were kept on ice until printing. MAGIC matrices with 0.5 wt% alginate mixed at a 1:1 added volume ratio to Matrigel were further mixed with a neutralized stock solution of 8.5 mg/mL rat tail collagen I (Advanced Biomatrix) to achieve 1 mg/mL collagen in MAGIC matrix. This matrix was used for printing at 4 °C before ECM cross-linking at 37 °C.

3.3 Human primary mammary organoid bioprinting and analysis: Deidentified normal, finite lifespan primary human mammary epithelial cells (HMEC) were provided by Drs. Martha Stampfer and James Garbe (Lawrence Berkeley National Laboratory). HMEC were cultured from tissues removed during reduction mammoplasties, and expanded to fourth passage in M87A medium as described previously[35]. HMEC from tissue donated by a 19 year-old individual with bilateral breast hypertrophy (240L) were used for all experiments. All HMEC were cultured in complete M87A medium with Penicillin-Streptomycin (100 U/mL) at 37 °C with CO^2 up to 80-90% confluency. Fourth passage HMEC were transduced with lentivirus upon thawing at a multiplicity of infection of 13 (to target 40-60% transduction efficiency) into a half volume of M87A medium containing 2 µg/ml polybrene (Millipore-Sigma #TR1003). After 3 hours, M87A medium was added to full volume. After 24-48h, the virus-containing medium was discarded and replaced with fresh M87A medium. Cells were grown up to 80-90% confluency (5-7 days). Transduced cells were isolated by FACS based on GFP or mCherry expression. To obtain a printable single-cell solution, 80-90% confluent HMEC were digested using TrypLE Express (Gibco) for 10 min at 37 °C. Cells were washed with D-PBS with 2 mM EDTA and filtered using a 40 µm cell strainer. GFP- or mCherry-expressing HMEC were sorted into myoepithelial and luminal populations and recombined into bioinks using 1:1 and 2:1 LEP:MEP or all-MEP compositions. Bioprinted HMEC organoids were analyzed for sorting one day after printing using ilastik[36] to segment based on fluorescence and custom Fiji scripts to assess boundary occupancy.

3.4 Human iPSC culture, cortical organoid differentiation, dissociation, and cell proportion analysis: iPSCs from three different donors were used (28126, 20916B, 13234)[37,38] were maintained on Matrigel-coated plates and cultured in StemFlex (Thermo Fisher #A3349401). To passage, cells were lifted with PBS without calcium or magnesium supplemented with 0.5 mM EDTA. For differentiation, iPSCs were lifted using PBS-EDTA and

resuspended in Neural Induction Media containing GMEM (Gibco #11710035), 10% Knockout Serum Replacement (Gibco #10828028), NEAA diluted 1:100 (Gibco #11140050), Sodium Pyruvate diluted 1:100 (Gibco #11360070), 5 mM 2-mercaptoethanol (Sigma Aldrich #M6250), and 100 µg/mL Primocin supplemented with 5 µM SB431542 (Tocris #1614), 100nM LDN-193189 (Sigma Aldrich #SML0559), and 3 µM IWR1-endo (Cayman Chemicals #13659). Cells in Neural induction media were moved to 6-well low attachment plates (Corning #3471), with a media change on day 3 with CEPT, and day 6 without CEPT. From day 9-25, organoids were cultured in Maintenance Media 1: 50% DMEM/F12 with Glutamax (Gibco #10565042) and 50% Neurobasal (Gibco #21103049) with B27 without vitamin A (Gibco #12587001), N2 (Gibco #17502048), NEAA diluted 1:100, Glutamax diluted 1:200 (Gibco #35050061), and 55 µM 2-mercaptoethanol supplemented with 10 ng/mL each FGF (Peprotech #100-18B) and EGF (Peprotech #100-47). Media was changed every 2-3 days. From days 26-35, media was changed without FGF and EGF. From day 35 onward, organoids were cultured in Maintenance Media 2: Maintenance Media 1 supplemented with B27 with vitamin A (Gibco #17504001), instead of B27 without vitamin A. For printing, organoids were dissociated on D49 using 20 units of Papain (Worthington #LK003178) with 5% Trehalose in HBSS for 30 minutes at 37 °C. DNase was added, and organoids were incubated for another 15 minutes at 37 °C. Papain was quenched using Albumin-ovomucoid inhibitor (Worthington # LK003182) and cells were filtered through a 40 µm cell strainer. Cells were spun down, resuspended, and counted.

For cell proportion quantification, 20 µM optical sections were used with maximum Z projection and LUT adjustment consistent for all images. CellProfiler[39] version 4.2.4 was used to quantify the number of cells positive for each protein of interest. Briefly, IdentifyPrimaryObjects was used to identify cells positive in each channel. Then RelateObjects and FilterObjects were used to assign a parent-child relationship between DAPI and each additional channel, removing cells with positive signal in one channel but not for DAPI. Percentage of cells positive for each marker were then calculated using these metrics.

3.5 Triple-negative breast cancer patient-derived organoid transduction & transfection: TORG139 patient-derived triple-negative breast cancer (TNBC) organoids were generated and cultured as previously described in type 1 mammary organoid medium[40–42]. To obtain a printable single-cell solution, organoids were digested using TrypLE Express (Gibco) for 10 min at 37 °C. Cells were washed in D-PBS with 2 mM EDTA and filtered using a 40 µm cell strainer. Single-cell solutions were kept on ice until printing. For transduction experiments, TNBC organoids were mechanically passaged and either incubated in suspension for 2 hours in media containing GFP-expressing lentivirus or replated in Matrigel or MAGIC matrix before adding media containing GFP-expressing lentivirus with a multiplicity of infection of 5 (10^6 viral particles per sample) and 2 µg/mL polybrene (Millipore-Sigma #TR1003). Bioprinted TNBC organoids were transduced in the same conditions with virus-containing media left on top of the matrix in a 96 well plate. Viral media was discarded 2 h (suspension) or 16 h (matrix-embedded and bioprinted) after transduction. Organoids were imaged at day 5 post-transduction and GFP expression was quantified by segmenting individual organoids and sub-segmenting those with GFP signal using ilastik[36]. Following manufacturer's instructions, bioprinted TNBC organoid arrays were transfected using Lipofectamine RNAiMAX (Thermo Fisher) in Type 2 medium[43] with 1% FBS and with 5 or 10 pmol of a Cy3-conjugated 36mer single-stranded non-coding small RNA (MW = 11.9 kDa) for 24, 48, or 72 h starting 3 days after printing. Images were taken with an Echo Resolve microscope.

3.6 Organoid Fixation and Immunofluorescent Staining: To preserve matrix structure, MAGIC matrices were pre-treated before fixation to hold bioprinted structures in place. Samples were first incubated for 20 min with 25 mM $CaCl_2$ in sterile milliQ H_2O to "lock" the alginate microgels. Then, a layer of warm liquid 0.5% agarose was poured over the samples and allowed to cool for 5 minutes at room temperature followed by 5 minutes at 4 °C. Samples were then fixed in 2% PFA for 45 min at room temperature and washed with PBS-glycine 3x, 20 min each followed by washing with PBS 2x, 20 min each. Samples were left overnight in 25 mM $CaCl_2$ solution, as long-term storage in PBS led to formation of insoluble calcium phosphate, which occluded the samples. The next day, fixed organoids were permeabilized with 0.5% Triton X-100 for 15 min at room temperature and blocked with blocking buffer for 2 h at room temperature or overnight at 4 °C. If using mouse-origin primary antibodies, samples were also incubated overnight with 1:50 dilution mouse Fab fragment (Jackson

ImmunoResearch #115-007-003). Samples were then incubated with primary antibody in blocking buffer for 24-48 h at 4 °C, rinsed in wash buffer 3 times for 1 h at room temperature, incubated with secondary antibody in blocking buffer overnight at 4 °C, and rinsed in wash buffer 3 times for 1 hr at room temperature. DAPI was added for 30 min and organoids were washed once more for 30 min in PBS. Finally, samples were cleared overnight in RapidClear 1.52 (SunJin Lab) at room temperature before imaging. Primary antibodies used include rat anti-ECCD2 (Thermo Fisher 131900), rabbit anti-LYZ (Thermo Fisher 129680), rabbit anti-CHGA (Novus Biologicals NB120-15160B), and chicken anti-GFP (Aves Labs GFP1010). Secondary antibodies were raised in goat and included Alexa Fluor 488 (Thermo Fisher A11039), Alex Fluor 568 (Thermo Fisher A11011), and Alexa Fluor 647 tags (Thermo Fisher A21247).

3.7 Microscopy: Live imaging of bioprinted structures during extrusion, which allowed for iterative human-in-the-loop adjustments to print parameters, was achieved using a Leica DMI8 programmed on the same custom GUI as the bioprinter components. After printing, live organoids were imaged using either a Zeiss Axio Observer Z1 with a Yokogawa spinning disk or GE Healthcare IN Cell Analyzer 2200 confocal microscope. Brightfield and live cell fluorescence (membrane tdTomato, green fluorescent protein) were captured using 5x/NA 0.25 or 10x/NA 0.3 air objectives in controlled environmental chambers held at 37 °C and 5% CO_2. Fixed organoids were imaged using a Zeiss LSM800 confocal microscope equipped with 20x/NA 0.8 LD air or 25x/NA 0.8 multi-immersion objectives. Image acquisition and stitching was controlled using ZEN 2.3 (2011) software. Subsequent image analysis, including background subtraction, filters, and thresholding was performed using custom macros in Fiji/ImageJ. Any image alterations, such as brightness & contrast adjustments or thresholding, were kept consistent across conditions in a given experiment for analysis.

4. References

1. Tee, S.-Y., Bausch, A. R. & Janmey, P. A. The mechanical cell. *Curr. Biol.* **19**, R745–R748 (2009).

2. Pérez-González, C. *et al.* Mechanical compartmentalization of the intestinal organoid enables crypt folding and collective cell migration. *Nat Cell Biol* **23**, 745–757 (2021).

3. Chaudhuri, O. *et al.* Hydrogels with tunable stress relaxation regulate stem cell fate and activity. *Nat Mater* **15**, 326–334 (2016).

4. Charrier, E. E., Pogoda, K., Wells, R. G. & Janmey, P. A. Control of cell morphology and differentiation by substrates with independently tunable elasticity and viscous dissipation. *Nat. Commun.* **9**, 449 (2018).

5. Oosten, A. S. G. van *et al.* Emergence of tissue-like mechanics from fibrous networks confined by close-packed cells. *Nature* **573**, 96–101 (2019).

6. Lee, H., Gu, L., Mooney, D. J., Levenston, M. E. & Chaudhuri, O. Mechanical confinement regulates cartilage matrix formation by chondrocytes. *Nat. Mater.* **16**, 1243–1251 (2017).

7. Nam, S., Stowers, R., Lou, J., Xia, Y. & Chaudhuri, O. Varying PEG density to control stress relaxation in alginate-PEG hydrogels for 3D cell culture studies. *Biomaterials* **200**, 15–24 (2019).

8. Indana, D., Agarwal, P., Bhutani, N. & Chaudhuri, O. Viscoelasticity and Adhesion Signaling in Biomaterials Control Human Pluripotent Stem Cell Morphogenesis in 3D Culture. *Adv. Mater.* **33**, e2101966 (2021).

9. Fan, W. *et al.* Matrix viscoelasticity promotes liver cancer progression in the pre-cirrhotic liver. *Nature* **626**, 635–642 (2024).

10. Borelli, A. N. *et al.* Stress Relaxation and Composition of Hydrazone-Crosslinked Hybrid Biopolymer-Synthetic Hydrogels Determine Spreading and Secretory Properties of MSCs. *Adv. Healthc. Mater.* **11**, e2200393 (2022).

11. Anis, Y., Houkal, J., Holl, M., Johnson, R. & Meldrum, D. Diaphragm pico-liter pump for single-cell manipulation. *Biomed. Microdevices* **13**, 651–659 (2011).

12. Zhu, J. & Cai, L.-H. All-Aqueous Printing of Viscoelastic Droplets in Yield-Stress Fluids. *Acta Biomater* (2022) doi:10.1016/j.actbio.2022.09.031.

13. Becker, M., Gurian, M., Schot, M. & Leijten, J. Aqueous Two-Phase Enabled Low Viscosity 3D (LoV3D) Bioprinting of Living Matter. *Adv. Sci.* **10**, 2204609 (2023).

14. Hijnen, N. & Clegg, P. S. Colloidal Aggregation in Mixtures of Partially Miscible Liquids by Shear-Induced Capillary Bridges. *Langmuir* **30**, 5763–5770 (2014).

15. Vasilyev, O. A., Labbé-Laurent, M., Dietrich, S. & Kondrat, S. Bridging transitions and capillary forces for colloids in a slit. *J. Chem. Phys.* **153**, 014901 (2020).

16. Hofer, M. & Lutolf, M. P. Engineering organoids. *Nat Rev Mater* **6**, 402–420 (2021).

17. Popova, G. *et al.* Human microglia states are conserved across experimental models and regulate neural stem cell responses in chimeric organoids. *Cell Stem Cell* **28**, 2153-2166.e6 (2021).

18. Shin, D. *et al.* Thalamocortical organoids enable in vitro modeling of 22q11.2 microdeletion associated with neuropsychiatric disorders. *Cell Stem Cell* **31**, 421-432.e8 (2024).

19. Chrisnandy, A., Blondel, D., Rezakhani, S., Broguiere, N. & Lutolf, M. P. Synthetic dynamic hydrogels promote degradation-independent in vitro organogenesis. *Nat Mater* 1–9 (2021) doi:10.1038/s41563-021-01136-7.

20. Nam, S., Lee, J., Brownfield, D. G. & Chaudhuri, O. Viscoplasticity Enables Mechanical Remodeling of Matrix by Cells. *Biophys. J.* **111**, 2296–2308 (2016).

21. Chrisnandy, A. & Lutolf, M. P. An extracellular matrix niche secreted by epithelial cells drives intestinal organoid formation. *Dev. Cell* (2025) doi:10.1016/j.devcel.2025.06.026.

22. Yavitt, F. M. *et al.* In situ modulation of intestinal organoid epithelial curvature through photoinduced viscoelasticity directs crypt morphogenesis. *Sci Adv* **9**, eadd5668 (2023).

23. Yavitt, F. M. *et al.* Engineered epithelial curvature controls Paneth cell localization in intestinal organoids. *Cell Biomater.* **1**, 100046 (2025).

24. Carvalho, E. M. *et al.* Viscoelastic High-Molecular-Weight Hyaluronic Acid Hydrogels Support Rapid Glioblastoma Cell Invasion with Leader–Follower Dynamics. *Adv. Mater.* e2404885 (2024) doi:10.1002/adma.202404885.

25. Qiao, E. *et al.* Spectrin mediates 3D-specific matrix stress-relaxation response in neural stem cell lineage commitment. *Sci. Adv.* **10**, eadk8232 (2024).

26. Roth, J. G. *et al.* Tunable hydrogel viscoelasticity modulates human neural maturation. *Sci. Adv.* **9**, eadh8313 (2023).

27. Nerger, B. A. *et al.* 3D Hydrogel Encapsulation Regulates Nephrogenesis in Kidney Organoids. *Adv. Mater.* e2308325 (2024) doi:10.1002/adma.202308325.

28. Marín-Llauradó, A. *et al.* Mapping mechanical stress in curved epithelia of designed size and shape. *Nat. Commun.* **14**, 4014 (2023).

29. Chaudhuri, O., Cooper-White, J., Janmey, P. A., Mooney, D. J. & Shenoy, V. B. Effects of extracellular matrix viscoelasticity on cellular behaviour. *Nature* **584**, 535–546 (2020).

30. Gjorevski, N. *et al.* Designer matrices for intestinal stem cell and organoid culture. *Nature* **539**, 560–564 (2016).

31. Claxton, N. L., Luse, M. A., Isakson, B. E. & Highley, C. B. Engineering Granular Hydrogels without Interparticle Cross-Linking to Support Multicellular Organization. *ACS Biomater. Sci. Eng.* **10**, 7594–7605 (2024).

32. Daly, A. C., Prendergast, M. E., Hughes, A. J. & Burdick, J. A. Bioprinting for the Biologist. *Cell* **184**, 18–32 (2021).

33. Ding, A. *et al.* Jammed Micro-Flake Hydrogel for Four-Dimensional Living Cell Bioprinting. *Adv. Mater.* **34**, 2109394 (2022).

34. Cabral, K. A. *et al.* Programming the Self-Organization of Endothelial Cells into Perfusable Microvasculature. *Tissue Eng Pt A* **29**, 80–92 (2023).

35. Stampfer, M. R., LaBarge, M. A. & Garbe, J. C. *Cell and Molecular Biology of Breast Cancer*. (Humana Totowa, New Jersey, 2013). doi:10.1007/978-1-62703-634-4.

36. Berg, S. *et al.* ilastik: interactive machine learning for (bio)image analysis. *Nat. Methods* **16**, 1226–1232 (2019).

37. Matsumoto, Y. *et al.* Induced pluripotent stem cells from patients with human fibrodysplasia ossificans progressiva show increased mineralization and cartilage formation. *Orphanet J. Rare Dis.* **8**, 190 (2013).

38. Romero, I. G. *et al.* A panel of induced pluripotent stem cells from chimpanzees: a resource for comparative functional genomics. *eLife* **4**, e07103 (2015).

39. Stirling, D. R. *et al.* CellProfiler 4: improvements in speed, utility and usability. *BMC Bioinform.* **22**, 433 (2021).

40. Sachs, N. *et al.* A Living Biobank of Breast Cancer Organoids Captures Disease Heterogeneity. *Cell* **172**, 373-386.e10 (2018).

41. Rosenbluth, J. M. *et al.* Organoid cultures from normal and cancer-prone human breast tissues preserve complex epithelial lineages. *Nat. Commun.* **11**, 1711 (2020).

42. Cho, N. W. *et al.* T cells Instruct Immune Checkpoint Inhibitor Therapy Resistance in Tumors Responsive to IL-1 and TNFα Inflammation. *bioRxiv* 2022.09.20.508732 (2024) doi:10.1101/2022.09.20.508732.

43. Dekkers, J. F. *et al.* Long-term culture, genetic manipulation and xenotransplantation of human normal and breast cancer organoids. *Nat. Protoc.* **16**, 1936–1965 (2021).
